# From aerial drone to quantitative trait locus: leveraging next‐generation phenotyping to reveal the genetics of color and height in field‐grown *Lactuca sativa*

**DOI:** 10.1111/tpj.70405

**Published:** 2025-08-13

**Authors:** Rens Dijkhuizen, Abraham L. van Eijnatten, Sarah L. Mehrem, Esther van den Bergh, Jelmer van Lieshout, Kiki Spaninks, Steven Kaandorp, Remko Offringa, Marcel Proveniers, Guido van den Ackerveken, Basten L. Snoek

**Affiliations:** ^1^ Theoretical Biology and Bioinformatics, Department of Biology, Science Faculty Institute of Biodynamics and Biocomplexity, Utrecht University Padualaan 8 Utrecht 3584CH The Netherlands; ^2^ Translational Plant Biology, Department of Biology, Science Faculty Institute of Environmental Biology, Utrecht University Padualaan 8 Utrecht 3584CH The Netherlands; ^3^ Plant Developmental Genetics Institute of Biology Leiden, Leiden University Sylviusweg 72 Leiden 2333 BE The Netherlands; ^4^ Bejo Zaden B.V. Trambaan 1 Warmenhuizen 1749 CZ The Netherlands

**Keywords:** lettuce, *Lactuca sativa*, drone, unmanned aerial vehicle, genome‐wide association study, natural variation, color, height

## Abstract

In recent years, accurate and low‐cost variant calling has enabled the genotyping of large diversity panels for genome‐wide association studies. As a result, phenotyping rather than genotyping is now the rate‐limiting step, especially in field experiments. This has created a strong need for high‐throughput, accurate, and low‐cost in‐field phenotyping. Here, we present a genome‐wide association study (GWAS) study on 194 field‐grown accessions of lettuce (*Lactuca sativa*). These accessions were non‐destructively phenotyped at two time points 15 days apart using a drone equipped with an RGB and multispectral (MSP) camera. Our high‐throughput phenotyping approach integrates an RGB‐ and MSP camera to measure the color and height of lettuce in this large‐scale field experiment. We used the mean and other summary statistics, such as median, quantiles, skewness, kurtosis, minimum, and maximum to quantify different aspects of color and height variation in lettuce from the drone images. Using these summary statistics as traits for GWAS, we confirm several previously described genetic associations, now under field conditions, and identify additional novel associations for color and height traits in lettuce.

## Significance Statement

Understanding the genetic basis of lettuce color is important because these traits correlate with metabolites such as anthocyanins, which are known to have strong antioxidant properties. Furthermore, the color of lettuce strongly affects consumer appeal. The height of lettuce is also an important trait because it can serve as a proxy for the bolting time. After bolting, lettuce turns bitter and loses market value. Using drone‐based imaging to phenotype 194 lettuce accessions at two time points, we identify several known and novel genetic associations with color and height traits and provide valuable methodological and data resources for future drone phenotyping field experiments and lettuce genetics studies.

## INTRODUCTION

Lettuce (*Lactuca sativa* L.) is one of the most popular salad vegetables worldwide and is an excellent source of dietary minerals, fibers, and phytochemicals with related health benefits (Shi et al., 2022). Many different lettuce accessions are grown worldwide, and new accessions are continuously introduced (Wei et al., 2021; Zhang et al., 2017). Lettuce accessions can be grouped into several genetically and phenotypically distinguishable morphology types, such as butterhead, cos, latin, cutting, stalk, crisp, and oilseed (Wei et al., 2021). Significant heritable phenotypic variation exists both within and between morphology types. For example, the leaves of different lettuce accessions exhibit a range of colors from green to red or purple (Falcioni et al., 2023; Su et al., 2020).

Precise phenotyping of color traits in field‐grown lettuce and the study of underlying genetic pathways has important implications for consumer health (Falcioni et al., 2022). Furthermore, an appealing color is a major factor in positive consumer perception of lettuce (Chonpracha et al., 2020). Variation in the greenness of lettuce is partly determined by chlorophyll content. The chlorophyll content has implications for the nutritional state and quality of leafy vegetables (Falcioni et al., 2023; Heimler et al., 2007). Previously, variation in chlorophyll content in lettuce has been linked to the transcription factor LsGLK (GOLDEN2‐like) (Zhang et al., 2022). GLK transcription factors play a central role in chloroplast biogenesis and the regulation of photosynthesis‐associated genes (Hernández‐Verdeja & Lundgren, 2024). Additionally, anthocyanins are responsible for the red or purple coloration displayed by many lettuce accessions. Anthocyanins and other flavonoids are strongly correlated with antioxidant activity. Red lettuce accessions are rich in these flavonoids and other bioactive compounds associated with health benefits (Cheng et al., 2014; Mampholo et al., 2016). Several genes such as *Red Lettuce Leaves* 1–4 (*RLL1, RLL2*, *RLL3*, *RLL4*), anthocyanidin synthase (*ANS*), and others have been implicated in lettuce anthocyanin content (Su et al., 2020; Zhang et al., 2017).

RGB and multispectral (MSP) cameras can be used to approximate chlorophyll and anthocyanin content. A high anthocyanin content typically results in a purple or red color. Chlorophyll content can be inferred using several established vegetation indices. Vegetation indices are ratios of specific wavelengths, designed to estimate physiological or biochemical plant traits (Falcioni et al., 2023; Huang et al., 2021; Kureel et al., 2022). An example is the well‐known normalized difference vegetation index (NDVI) which was initially developed to estimate plant cover from satellite data (Rouse et al., 1973).

Usually, only the mean color intensity or vegetation index value is used to calculate per genotype traits. However, mean or median color traits are insensitive to spatial variation. For example, the mean red intensity of a green leaf with some red speckles and a homogeneous slightly red leaf will be the same. To capture this spatial heterogeneity, we also use alternative summary statistics of color intensities and vegetation indices, such as quantiles, minimum, and maximum.

Another commercially important trait for lettuce is bolting time. Bolting is the transition from the vegetative growth stage to the reproductive growth stage, marked by rapid elongation of the stem (Chen et al., 2019). Because bolting makes the plant taste bitter, lettuce is typically harvested in its vegetative growth state. Therefore, delayed bolting and stable flowering time are desirable traits in lettuce (Leijten et al., 2018). Bolting and flowering time are complex traits that have been extensively researched. Both are influenced by ambient temperature, photoperiod, the gibberellic acid pathway, age, and carbohydrate status (Han, Truco, et al., 2021). A total of 67 non‐overlapping QTLs have been reported for bolting and flowering time (Han, Truco, et al., 2021). Bolting time in lettuce is strongly affected by a lettuce homolog of the Arabidopsis gene *Phytochrome C* (Chen et al., 2025; Han, Truco, et al., 2021; Lee et al., 2021; Rosental et al., 2021).

Phenotypic variation can be associated with potential underlying genomic loci using a statistical procedure called genome‐wide association study (GWAS). Genetic loci associated with trait variation are referred to as quantitative trait loci (QTLs). Due to the advancement of next‐generation sequencing, the bottleneck for GWAS is no longer genotyping but phenotyping (Spindel et al., 2018; Watanabe et al., 2017; Xiao et al., 2022). Recently, several studies have increased phenotyping efficiency by applying modern drone and sensor technology (Ding et al., 2023; Han, Wong, et al., 2021; Spindel et al., 2018; Ye et al., 2023). Drone‐assisted measurements provide an opportunity for large‐scale in‐field phenotyping.

In this study, we performed GWAS on plant color and height traits of 194 *L. sativa* accessions grown in a large field experiment. The traits used for GWAS were obtained using an aerial drone, which imaged the field at two time points 15 days apart using an RGB and a MSP camera. Furthermore, a 2.5‐dimension (2.5D) approach was used to determine plant height. The genotypes of the lettuce accessions were obtained using the assemblies from Wei et al. (2021) combined with 67 additionally sequenced lines (Van Workum, Mehrem, et al., 2024). We show that the measured traits form coherent clusters with a shared genetic basis. Our approach is validated by detecting previously detected QTLs. We recover QTLs that were previously mapped in field conditions around the lettuce genes *PhyC*, *RLL2*, and *ANS* (Wei et al., 2021), and we recover QTLs that were previously only detected in lab conditions around the lettuce genes *RLL3*, *RLL4*, *GST*, and *LsGLK* (Rosental et al., 2021; Su et al., 2020; Zhang et al., 2017, 2022, 2023). Furthermore, we report multiple, to the best of our knowledge, novel QTLs affecting variation in color and height, demonstrating the power of drone‐assisted phenotyping. Some of the QTLs were found on only one of the two imaging days, showing that QTLs can depend on the growth stage of lettuce. We also show the added value of constructing additional traits as ratios from the imaging data and describing phenotypes with not only the mean but also additional summary statistics (extended descriptives) such as quantiles or the standard deviation. Taken together, this study shows how to effectively use drone‐assisted phenotyping to reveal the genetics of color and height under field conditions in an important crop species.

## RESULTS

### Phenotypic variation is highly heritable

To investigate the genetic basis of color and height traits in lettuce, we grew 194 lettuce accessions (Table S1) in a field near the nature area “de Peel” in the Netherlands (Figure S1). All data were obtained with the help of an aerial drone. Phenotyping was done on the 11th of June 2021 (78 days after sowing) and on the 25th of June 2021 (93 days after sowing). There was a considerable amount of rain in the 2 weeks between the first and second measurement days, as well as fluctuations in temperature (weather data available on https://doi.org/10.24416/UU01‐S5FCM9), potentially impacting plant phenotypes. We quantified 75 traits from the images taken by the drone (Table S4). These traits consist of the RGB and MSP values, several vegetation indices, and height. We also used several color ratios as traits (Table S3).

**Figure 1:**
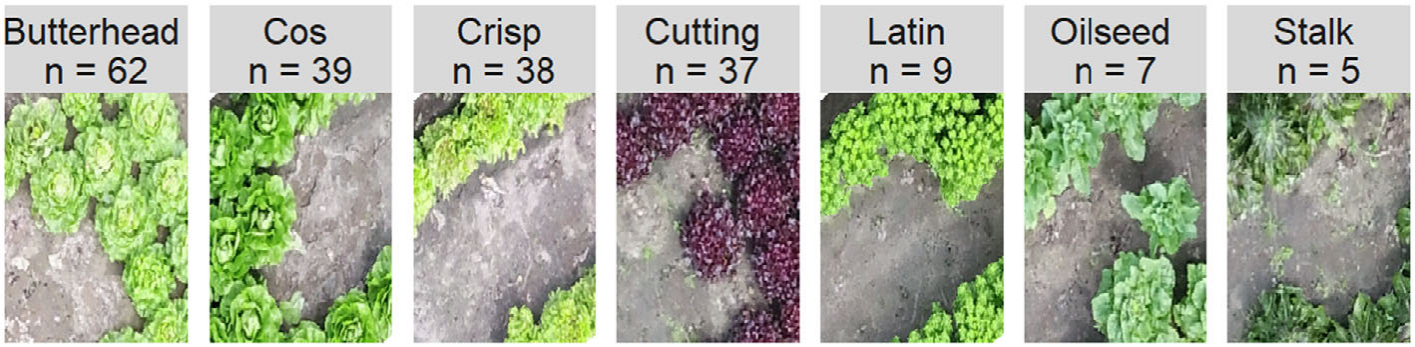
Seven different types of lettuce from the perspective of the unmanned aerial vehicle. One accession per lettuce morphology type is shown. The name of the lettuce morphology type is shown on top of each panel. The number of accessions per morphology type used in this study is represented by *n* on top of each panel.

**Figure 2:**
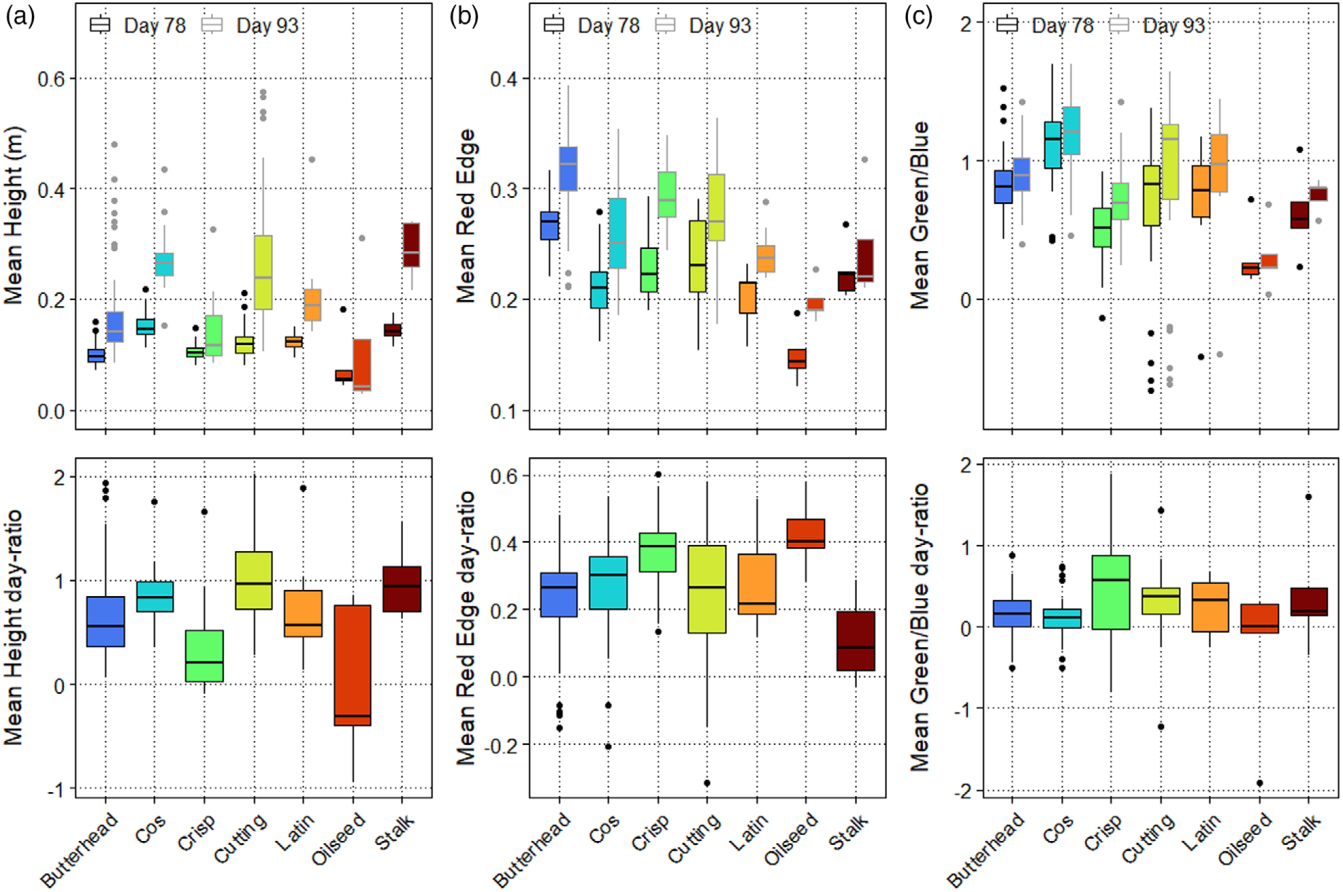
Variation in selected traits between seven lettuce types over 2 days. (a) Mean height of seven lettuce types over the 2 days. The seven lettuce morphology types are butterhead (*N* = 62), cos (*N* = 39), crisp (*N* = 39), cutting (*N* = 37), latin (*N* = 9), oilseed (*N* = 7), stalk (*N* = 5). (b) Mean red‐edge values from the multispectral camera. (c) Mean green/blue ratio from the RGB camera. Upper panels show the values at Day 78 and 93, and lower panels show the day‐ratio. Lettuce morphology types are indicated on the *x*‐axis and by different colors.

Substantial variation in these 75 traits was observed across seven lettuce morphology types present in our population, both within and between days (Figures 1 and 2; Figures S10 and S11). For example, we noticed that the cos, cutting, and stalk lettuce types had the largest increase in height between the two measuring days, whereas the oilseed type had the smallest increase (Figure 2a). The red‐edge trait, which describes the reflection on the edge between visible and infra‐red light, was found to increase from Day 78 to 93. Oilseed types had the lowest red‐edge values, whereas butterhead types had the highest values, especially at Day 78 (Figure 2b). The green/blue ratio was found to increase from Day 78 to 93 for almost all accessions. Since the purple‐colored accessions (mostly found in cutting lettuce) reflect more blue than green light, they have negative values for this trait (Figure 2c). In summary, the lettuce population shows substantial variation within and between measurement days and within and between lettuce morphology types.

Most of the phenotypic variation was found to be highly heritable (Figure S12; Table S10). The average heritability on Day 78 and 93 is 82.7% (standard deviation [SD] = 18.4) and 84.1% (SD = 18.5), respectively. The day‐ratio heritability was less heritable, with an average heritability of 61.4% (SD = 25.1). The minimum and skewedness had the lowest heritabilities out of the different trait descriptions. In contrast, the high quantiles often show increased heritability compared with the mean trait values (Figure S13). Overall, these 194 lettuce accessions displayed considerable heritable phenotypic variation, enabling investigation of the genetic architecture of these traits.

### Correlation between traits indicates a shared genetic architecture

**Figure 3:**
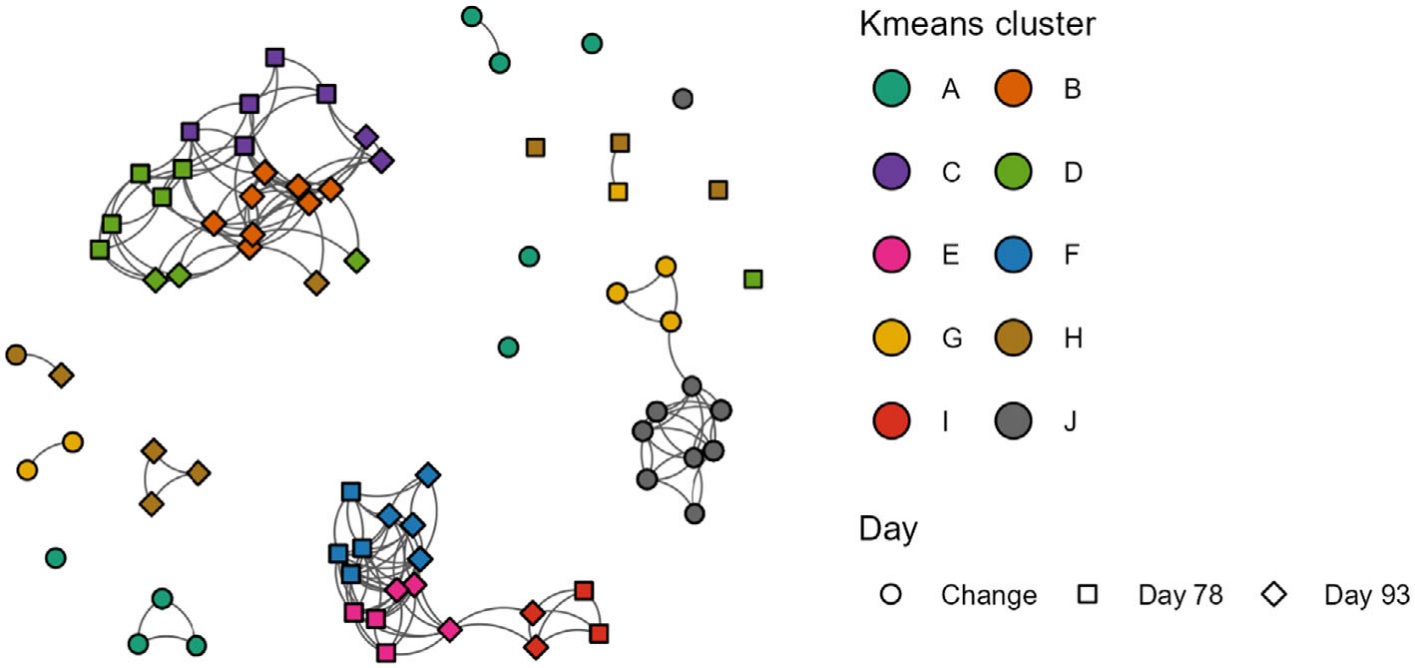
Visualization of correlation between traits using clusters obtained using K‐means clustering. K‐means clustering on all mean traits. Each node represents a trait, and each edge represents a correlation ≥0.8 between traits. Traits from Day 78, 93, and day‐ratio are shown by different shapes. The K‐means clusters are indicated by different colors.

Traits that are highly correlated are redundant and would likely show the same QTLs after GWAS. To quantify the similarity between the traits, we calculated the Pearson correlation between all traits (Table S11). Many traits showed high correlation, such as msp1 at Day 78 and msp3 at Day 78 (Pearson correlation = 0.93). To visualize this, we constructed a correlation network with traits as nodes and correlations exceeding 0.8 as edges (Figure 3). Most (68 of 75) of the traits share edges and can be found in clusters. The rest of the traits (7 of 75) are isolated from the rest of the network. Some just fall short of the correlation threshold. Others, such as the change in red/blue ratio, are not correlated to any of the other traits (Table S11; Figure S14). We used K‐means, an unsupervised clustering method, to divide the network into 10 clusters based on the squared correlation matrix of the traits (Figure 3). In general, traits from Day 78, 93, and especially the traits describing the day‐ratio tend to correlate more within their respective groups than between groups. We also saw that cluster E, cluster F, and cluster I, all consisting of ratios of RGB values, were connected. Other large clusters were cluster B, formed entirely by color ratio traits; cluster C, consisting mostly of several vegetation indices aimed at quantifying chlorophyll content; and cluster D, formed by several traits relating to the greenness of the plant. Clusters of traits likely indicate that these traits share part of their genetic architecture and are partial descriptions of a more complex phenotype. Due to the clusters of correlating traits, we expected to find several shared loci using GWAS for traits in the same cluster, as opposed to a unique SNP association pattern for each individual trait.

### Genome‐wide association studies show multiple known and novel QTLs

**Figure 4:**
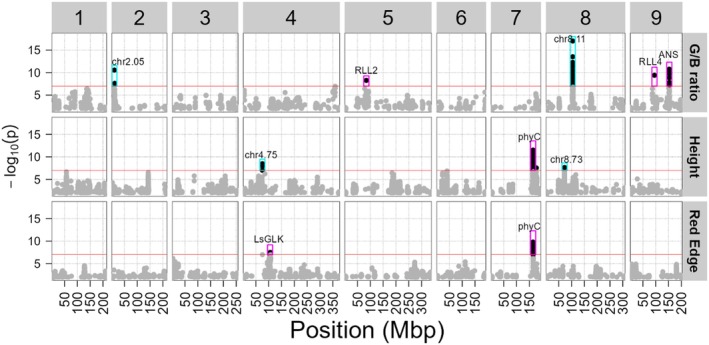
Manhattan plots for G/B ratio, height, and red edge on Day 93 show multiple QTLs. The *x*‐axis represents the genome of *L. sativa* in megabasepairs (Mbp). Chromosomes are shown on top. The *y*‐axis represents the *P*‐value of the association between the SNPs and the specific trait. The red line at −log_10_(*P*‐value) = 7 represents the significance threshold. SNPs above this threshold are shown in black, and SNPs below are shown in gray. The purple rectangles and their labels highlight QTLs and candidate genes that have been previously reported. The teal rectangles highlight QTLs that, to the best of our knowledge, have not been previously reported, and their labels indicate the QTL chromosome and position in Mbp.

To uncover the polymorphic loci underlying the observed phenotypic variation, we performed single nucleotide polymorphism (SNP)‐based GWAS for all 75 mean traits. We generated a genetic map with 1 154 639 SNPs, which showed a median linkage decay (*r* ^2^ ≤ 0.8) of 371 kbp in the population used in this study (Table S12; Figure S15). Using GWAS we found significantly associated SNPs [−log_10_(*P*) > 7] for 49 traits (Table S13). As examples we show the GWAS results for the Day 93 Green/blue ratio, Red Edge, and Height traits (Figure 4; Tables S14–S16). The green/blue ratio describes whether the color is more green or more purple. This is a good proxy for anthocyanin content (Figure S10). For this trait, we recovered *RLL2* (chr 5, 84.5 megabasepairs [Mbp]), *RLL4* (chr 9, 94.9 Mbp) and ANS (chr 9, 151.7 Mbp). All these loci are essential to the typical purple color seen in some lettuce accessions (Su et al., 2020). For the variation in height at Day 93, we found a QTL on chromosome 7 at 164.3 Mbp. The QTL locus contained the previously identified phytochrome C locus associated with bolting and flowering time in lettuce (Rosental et al., 2021). This QTL is not detected when using the height at Day 78 as a trait for GWAS (Figure S16). This result suggests that a substantial number of lettuce accessions started bolting between the first and second day of phenotyping. We also found, to the best of our knowledge for the first time, QTLs for the height at Day 93 on chromosome 4 at 75.4 Mbp and chromosome 8 at 72.9 Mbp (Figure 4). When looking at the red‐edge intensity, we find a QTL at the “pale leaf locus” (chr 4, 105.0 Mbp). This locus harbors the *LsGLK* gene, which is involved in variation in chlorophyl content (Zhang et al., 2022). Recovering these known loci solidifies the validity of our phenotyping and data‐analysis approach.

**Figure 5:**
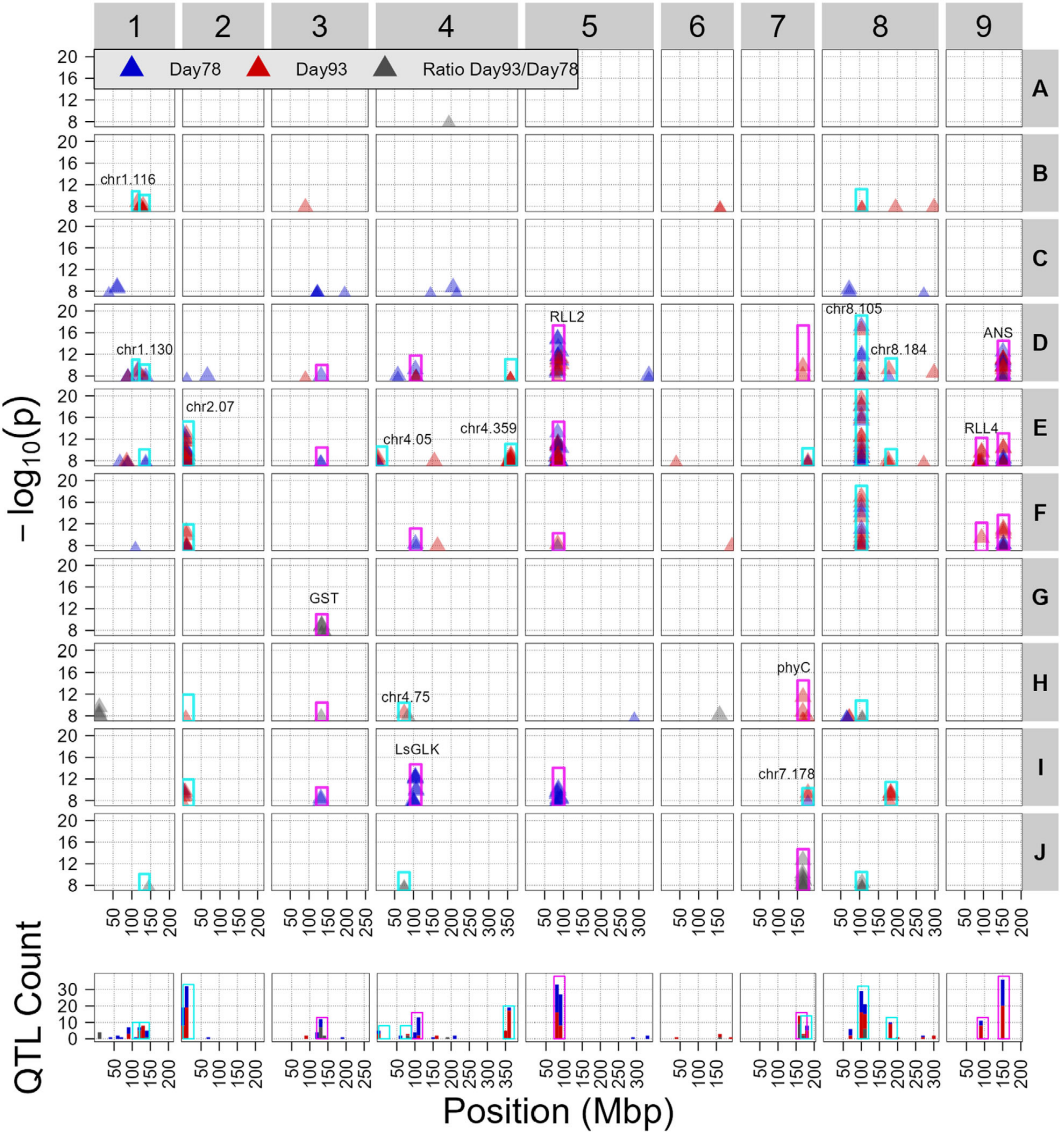
Multi‐Manhattan plot of all trait clusters. Every row represents one of the clusters (Table 1; Figure 3), shown on the right. The *y*‐axis represents the −log_10_(*P*‐value) the peak SNPs per trait per QTL. Loci that have been previously reported in the literature (Table 2) are highlighted with a purple rectangle and new loci with a cyan rectangle. For overlapping QTLs, only the most significant was highlighted. The color of the points represents whether the traits are from Day 78 (blue), Day 93 (red), or the difference between the 2 days (gray). The *x*‐axis represents the position on the genome of *L. sativa* in megabase pairs (Mbp). At the bottom, a histogram depicts the total amount of significant [−log_10_(*P*) > 7] in a 10 Mbp window. The color of the bars shows what day the traits are from.

To visualize the combined GWAS results of all 49 traits with significant SNPs, we took the lowest *P*‐value per 1 Mbp genomic bin and plotted all bins with [−log_10_(*P*) > 7] in one figure (Figure 5; Table S13), showing each cluster separately. Robust QTLs were selected by taking all loci where at least three unique traits have a −log_10_(*P*) score >7 within a 2.5 Mbp window (Table 2; Table S8; cyan and purple rectangles in Figure 5). We hypothesized that traits that clustered together based on correlations would show a similar QTL pattern. Our results substantiate this hypothesis, since every cluster has its own pattern of detected QTLs. Furthermore, clusters that grouped together in the correlation network (Figure 3) also had overlapping QTLs. For example, clusters F, E, and I grouped together based on correlations and showed similar GWAS results. Cluster D was an exception, correlating mostly with clusters B and C but showing similar GWAS results to clusters E and F. Many QTLs were recovered by several traits from different clusters, such as the QTLs containing *RLL2*, *RLL4*, *ANS*, and *PhyC*. This aligns with expectations as many of the color traits show overlap and have a high correlation.

**Table 1:** Contents of trait clusters

Cluster	Traits
A	Day‐ratio: $\frac{\text{green}}{\text{total color}}$, $\frac{\text{blue}}{\text{total color}}$, $\frac{\text{green}+\text{blue}}{\mathrm{red}}$, $\frac{\text{green}}{\text{blue}}$, $\frac{\mathrm{red}}{\text{blue}}$, $\frac{\mathrm{red}}{\text{total color}}$, $\frac{\mathrm{red}+\text{blue}}{\text{green}}$, $\frac{\mathrm{red}+\text{green}}{\text{blue}}$, $\frac{\mathrm{red}}{\text{green}}$
B	Day‐93: total color, msp1, red, CIred, NDVI, msp3, NDRE, SR
C	Day‐78: SR, msp3, NDVI, ARVI, SIPI Day‐93: ARVI, SIPI
D	Day‐78: green, msp2, total color, red, msp1, msp4 Day‐93: green, msp2, msp4
E	Day‐78: $\frac{\text{green}}{\text{total color}}$, $\frac{\mathrm{red}+\text{blue}}{\text{green}}$, $\frac{\mathrm{red}}{\text{green}}$ Day‐93: $\frac{\text{green}}{\text{total color}}$, $\frac{\mathrm{red}+\text{blue}}{\text{green}}$, $\frac{\mathrm{red}}{\text{green}}$
F	Day‐78: $\frac{\text{blue}}{\text{total color}}$, $\frac{\text{green}}{\text{blue}}$, $\frac{\mathrm{red}}{\text{blue}}$, $\frac{\mathrm{red}+\text{green}}{\text{blue}}$ Day‐93: $\frac{\text{blue}}{\text{total color}}$, $\frac{\text{green}}{\text{blue}}$, $\frac{\mathrm{red}}{\text{blue}}$, $\frac{\mathrm{red}+\text{green}}{\text{blue}}$
G	Day‐78: msp5 Day‐ratio: EVI, ARVI, NDVI, msp5, SIPI
H	Day‐78: height, EVI, blue Day‐93: height, EVI, blue, msp5, WDVI Day‐ratio: height
I	Day‐78: $\frac{\text{green}+\text{blue}}{\mathrm{red}}$, $\frac{\mathrm{red}}{\text{total color}}$ Day‐93: $\frac{\text{green}+\text{blue}}{\mathrm{red}}$, $\frac{\mathrm{red}}{\text{total color}}$
J	Day‐ratio: msp1, msp2, msp3, msp4, red, green, blue, total color, SR

The Cluster column shows the cluster name and the Traits column shows the traits in each cluster.

**Table 2:** All robust QTLs using mean color and height traits

Chromosome	Position Mbp (v8)	Most significant trait	−log_10_ (*P*) max	Candidate gene^a^	Source candidate gene
1	115.65	Green Day 78	9.03		
1	129.88	Color total‐Day 93	8.13		
2	0.64	$\frac{\mathrm{Red}}{\text{Green}}$ Day 78	13.25		
2	10.53	$\frac{\mathrm{Red}+\text{Blue}}{\text{Green}}$ Day 93	12.92		
3	133.27	NIR day‐ratio	8.97	*GST*	Zhang et al. (2017)
4	4.94	$\frac{\mathrm{Red}}{\text{Green}}$ Day 93	8.60		
4	75.40	Height Day 93	8.48		
4	105.00	$\frac{\text{Green}+\text{Blue}}{\mathrm{Red}}$ Day 78	12.67	*LsGLK*	Zhang et al. (2022)
4	358.76	$\frac{\mathrm{Red}}{\text{Green}}$ Day 93	9.09		
5	84.50	Green Day 78	15.34	*MYB113/114 (RLL2)*	Su et al. (2020); Zhang et al. (2023)
7	164.25	Red Edge day‐ratio	12.69	*PhyC*	Rosental et al. (2021)
7	177.61	$\frac{\mathrm{Red}}{\text{total}}$ Day 93	9.40		
8	104.73	$\frac{\mathrm{Red}+\text{Blue}}{\text{Green}}$ Day 93	19.33		
8	183.01	$\frac{\mathrm{Red}}{\text{TotalColor}}$ Day 93	9.44		
9	94.93	$\frac{\mathrm{Red}+\text{Blue}}{\text{Green}}$ Day 93	10.22	*RUP1/2 (RLL4)*	Su et al. (2020)
9	151.73	Green Day 78	12.50	*ANS*	Zhang et al. (2017)

From all the GWAS results, we selected robust QTLs (all robust QTLs can be found in Table S8). To qualify as a robust QTL, at least three traits with a −log_10_(*P*) > 7 within a 2.5 Mbp window are required. We reported the statistics of the trait with the lowest *P*‐value mapping to robust QTLs. The first and second columns show the chromosome and position of the most significant SNP of the QTL peak in megabase pairs (Mbp) on the lettuce reference genome v8 (NCBI). The third column shows the trait with the lowest *P*‐value. The fourth column shows the *P*‐value. The fifth column shows candidate genes reported in the literature. The sixth column shows the source for the candidate gene.

^a^ For all candidate genes at the locus, see Table S8. This table includes several suggestions for the novel QTLs.

On top of the previously known QTLs, we also found several new QTLs that, to the best of our knowledge, have not been reported before in lettuce (Table 2). Strikingly, we found a novel QTL with high confidence [−log_10_(*P*) > 19] on chromosome 8 at 105 Mbp for the green/blue ratio and several other traits, which, to the best of our knowledge, has not yet been reported in the literature. Most accessions that are homozygous for the alternate allele at the most significant SNP show the typical purple color associated with anthocyanin (Figure S17). We noticed that the candidate gene *FQR1* (105.00 Mbp) is close to the most significant SNP (105.73 Mbp). This gene is described as a flavodoxin. Although there is no direct relation between flavodoxins and flavonoids, the FQR1 protein has been shown to affect pigmentation *in vitro* in Arabidopsis (Laskowski et al., 2002). We found another QTL on the same chromosome (chr8, 183.01 Mbp) for several traits relating to the green/red ratio (Table S13). We also found, to the best of our knowledge, not previously reported robust QTLs related to color on chromosome 1 (115.65 and 129.88 Mbp), chromosome 2 (0.64 Mbp), chromosome 4 (4.94 and 358.76 Mbp) and chromosome 7 (177.61 Mbp). In summary, our study uncovered many novel QTLs related to the color of lettuce under field conditions, possibly offering new breeding targets.

### Extended trait descriptives support and uncover more QTLs and lead to higher significance

**Figure 6:**
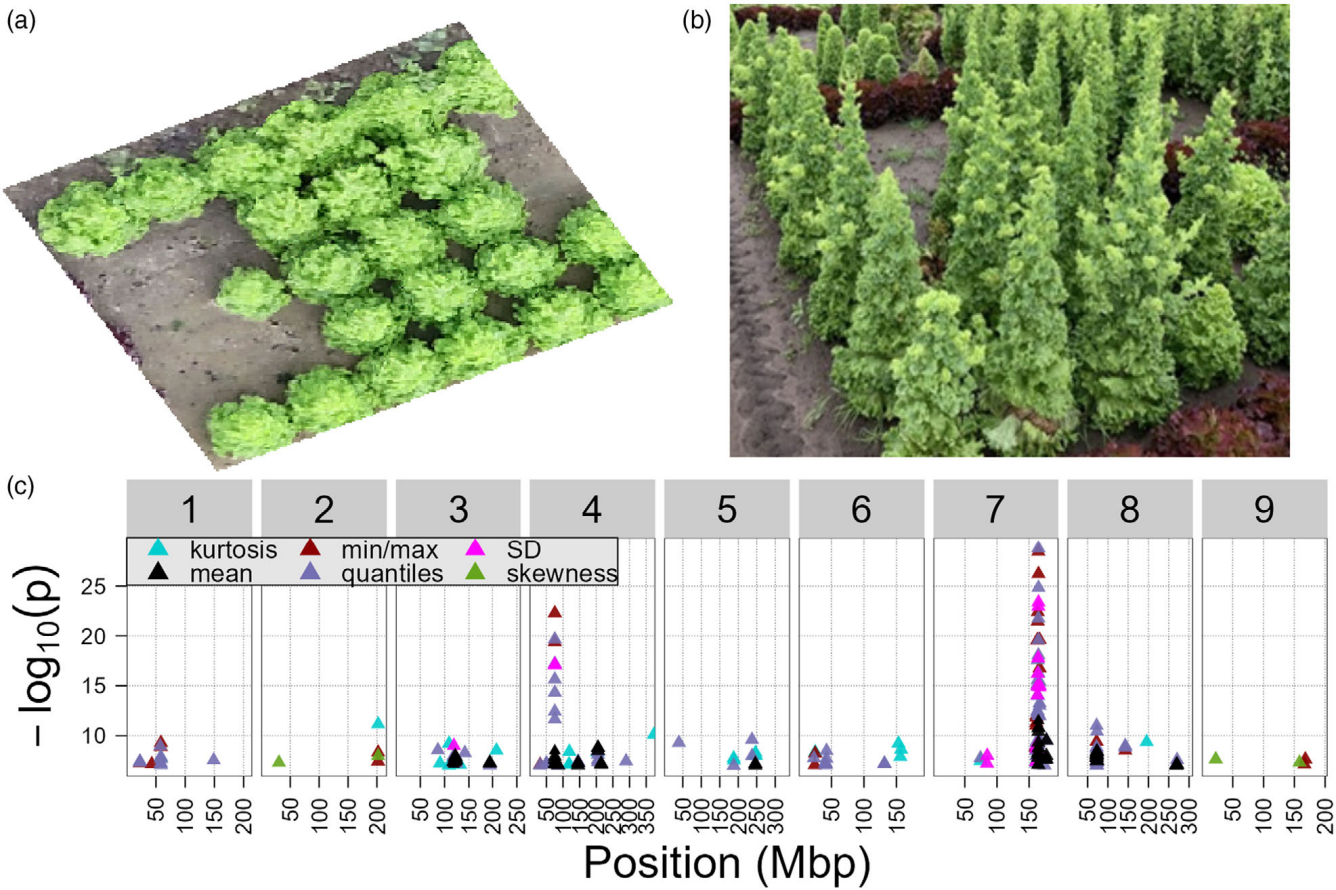
Extended descriptives and GWAS on height traits. (a) A lettuce accession bolting as seen from a top perspective. (b) A bolting lettuce accession as seen from a side perspective. (c) GWAS results on all extended descriptive height traits. The middle of the triangle indicates the position of the QTL. Black triangles show the results of the GWAS on the mean traits and in colors several extended descriptives. All details can be found in Table S17.

In addition to analyzing the mean of the traits, we also investigated several extended descriptives to describe different aspects of certain phenotypes, potentially yielding distinct GWAS results. The extended descriptives we used are: trimmed means, several quantiles, the median, the standard deviation, the minimum and maximum value, the skewness, and kurtosis of the trait distribution (Table S2). To test the usefulness of these descriptives, we compared them to the GWAS results obtained using only mean trait values. We hypothesized that these descriptives are more sensitive to specific phenotypes than the mean trait value. For example, when a lettuce plant starts bolting, this should have a much stronger effect on the maximum height of the plant than the average height over all plant parts (Figure 6a,b). To test this, we compared the results of all mean height traits with their extended descriptives (Figure 6c; Table S17).

We found that the extended descriptives sometimes represented the traits better than the mean, leading to higher significance in GWAS. For example, the mean height on Day 93 mapped to the *PhyC* locus with −log_10_(*P*) = 11.5, yet the maximum height on Day 93 maps to the same locus with −log_10_(*P*) = 26.2 (Figure 6c). The most significant trait was the 95% quantile day‐ratio of height with a −log_10_(*P*) = 28.1. For the height on Day 93 QTL on chromosome 4 at 75.4 Mbp the significance increased from −log_10_(*P*) = 8.5 with the mean height to −log_10_(*P*) = 19.4 with the maximum height. Similarly, the maximum and quantiles of height on Day 93 had a QTL on chromosome 1 58.4 Mbp with −log_10_(*P*) = 9.3. The mean height barely missed the significance threshold with −log_10_(*P*) = 6.7 (Figure 6c; Tables S8, S9, and S17).

Following the same strategy of clustering traits, we performed a new K‐means clustering, combining the mean traits with all extended descriptives (Figures S9 and S18). The clustering on all traits was remarkably similar to the clustering on only mean traits. In this new clustering, we also found a cluster containing most height traits (cluster B, all traits), a cluster containing traits related to the redness of plants (cluster F, all traits), and a cluster containing traits related to the greenness of plants (cluster G, all traits) (Figure S18). Most QTLs found with the extended descriptives were also found with the mean traits (Figure S9). However, using extended descriptives, we found the previously reported *RLL3* locus (Su et al., 2020), which we did not find using only mean traits (Figure S9; Table S9). This locus was recovered by the maximum green trait and several quantiles of the green/blue ratio and red/green ratio. This shows that using quantiles and maxima is beneficial for identifying QTLs not well described by mean traits. For other QTLs, using extended descriptives as traits in GWAS often resulted in detecting these shared QTLs with a higher significance (Figure S9). While the quantiles and maximum often led to higher significance compared with mean traits, the standard deviation, skewness, and kurtosis appear to give unreliable results, as these descriptives have a low heritability and fail to uncover well‐defined QTLs (Figures S13 and S19). In summary, some extended descriptives are often better trait descriptions compared with the mean, leading to higher confidence mapping and additional QTLs.

## DISCUSSION

In recent years, advancements in genotyping technologies have alleviated the bottleneck previously posed in GWAS studies (Spindel et al., 2018; Watanabe et al., 2017). Now, phenotyping is often considered to be the limiting step, especially in field conditions. In this study, we grew 194 lettuce (*L. sativa*) accessions in a field setting and employed drone‐assisted imaging and automatic phenotyping to unravel the genetic underpinnings of color and height traits. Our GWAS uncovered several previously known QTLs and several new QTLs associated with these traits. By using descriptives other than the mean such as quantiles and maximum, we detected many loci with higher confidence and uncovered new loci. In addition, we showed that by using remote sensing for non‐destructive phenotyping over multiple days, we can uncover genetic loci that have a different association with trait variation in one developmental stage compared with another.

### Previously discovered QTLs

Multiple previous studies have investigated the genetic background of color and height traits in lettuce (Falcioni et al., 2023; Rosental et al., 2021; Su et al., 2020; Tripodi et al., 2023; Wei et al., 2021). Thus, some of the QTLs we found have been discovered before. Our analysis identified QTLs at *RLL2*, *RLL3*, and *RLL4* (Su et al., 2020; Zhang et al., 2023) and *ANS*, the latter being responsible for the second last step during anthocyanin synthesis (Wilmouth et al., 2002; Zhang et al., 2017). These QTLs are associated with several traits describing the purple color in the plant, which is a proxy for anthocyanin content. We also uncover a QTL containing *LsGLK*, a gene that plays a pivotal role in chloroplast development. This gene was previously shown to be vital for the typical green color present in many lettuce accessions (Chen et al., 2016; Zhang et al., 2022). We also identify a QTL containing the well‐known bolting gene *Phytochrome C* (Chen et al., 2025; Rosental et al., 2021). Of these QTLs, only the QTLs around *PhyC*, *RLL2*, and *ANS* have been previously identified under field conditions (Wei et al., 2021). There are a few possible explanations, apart from different phenotyping approaches, for the fact that our study finds additional QTLs compared with the field study by Wei et al. (2021). One explanation is a difference in population composition and size, causing additional segregating alleles affecting color and height traits. Another possible reason is that many QTLs are only found at specific developmental stages or in certain environmental conditions (Bac‐Molenaar et al., 2016; van Eijnatten et al., 2024; Vasseur et al., 2014). The study from Wei et al. (2021), which measured only one time point, may have missed some QTLs whose effects depend on the developmental stage. This might also explain why we do not find a QTL containing *RLL1*, a gene that was previously shown to affect anthocyanin content and color traits (Su et al., 2020). A final explanation for why we discover more QTLs than Wei et al. (2021) is the use of extended descriptives, which helps capture different facets of phenotypic variation. We would not have been able to detect the QTL containing *RLL3* in this experiment using only the mean traits.

### Novel QTLs

In addition to uncovering several previously reported QTLs, our analysis uncovered novel QTLs. For several novel QTLs, we identified candidate genes by examining the Arabidopsis homologs near the QTLs (Table S8). On chromosome 4 at 75.4 Mbp, we find a novel QTL associated with height on Day 93 and height day‐ratio. Many of these traits also map to *Phytochrome C* (Figure 4; Figure S16; Table S9). Close to this QTL are two potential candidate genes. The closest candidate gene to the top SNP (76.2 Mbp) is a homolog of *VOZ1*, a gene known to promote the flowering transition in Arabidopsis (Celesnik et al., 2013). Another potential candidate located at 77.1 Mbp is a homolog of *SPL3*, also known to affect flowering transition in Arabidopsis (Yamaguchi et al., 2009). This QTL on chromosome 4 related to height traits potentially overlaps with a previous QTL identified for flowering time in a RIL population (Han, Truco, et al., 2021).

We identified a QTL on chromosome 8 at 104.7 Mbp with high confidence (Figure 5). This QTL is mostly linked to traits relating to the green/blue ratio on Day 93. Examining the allele distribution of the most significant SNP in this QTL, we noticed that five out of nine genotypes that are homozygous for the alternative allele show the typical purple anthocyanin color. These five genotypes are the genotypes with the lowest green/blue ratio, which translates to the most purple (Figure S17). This is a clear indication that this allele is involved in anthocyanin distribution, since only around six (Figure S10) of all 194 genotypes are this color, with some additional accessions that have spotted leaves or different colors between the inner and outer leaves. A potential candidate gene for this QTL might be the homolog of the Arabidopsis gene *FQR1* at 105 Mbp. *FQR1* encodes a flavodoxin that has been shown to affect pigmentation *in vitro* (Laskowski et al., 2002). Another candidate gene for this QTL is a homolog of the R2R3 MYB transcription factor MYB119. This transcription factor is known to be involved in the regulation of anthocyanin production in Arabidopsis (Cho et al., 2016).

### Two measurement days show developmental stage specific QTLs

We conducted imaging at 78 and 93 days old. We imaged twice because we expected different QTLs/genes to be relevant depending on the developmental stage of the plant. Time‐dependent QTLs have been observed in other studies for various plant traits including flowering time, height, and senescence in potato (Hurtado‐Lopez et al., 2015), plant height in oilseed rape (Wang et al., 2015), and root architecture in maize (Cai et al., 2012). We found that some lettuce color and height QTLs are also time‐dependent. For example, the QTL containing the *LsGLK* gene (Zhang et al., 2022) is almost exclusively found with traits from Day 78, perhaps indicating a switch from resource investment in photosynthesis to resource investment in flowering between Day 78 and 93. In congruence with this hypothesis, the QTL containing the bolting regulator *PhyC* (Rosental et al., 2021) is exclusively found with traits from Day 93 and the ratio between 2 days. Interestingly, the *PhyC* locus is also found with traits like the red‐edge reflection, suggesting that bolting affects the color intensity measured by drone cameras. The day‐ratio traits were especially useful in uncovering the PhyC locus. Furthermore, the Day 93 allowed us to find a QTL on chromosome 4 at 358.8 Mbp that we would have otherwise missed. These results show that traits are impacted by different loci based on the growth stage of the plant. This observation is especially striking since the two imaging days are only 15 days apart, which is a relatively short time span for lettuce growth and development. With the rise of automatic phenotyping, this kind of multi‐day, multi‐phenotype data is likely to become more abundant. This will enable the discovery of more developmental stage‐dependent QTLs in the near future.

### Environment

Apart from variation in development, changes in the environment can also cause QTL variation. The plants in this experiment were transplanted outside on the 27th and 28th of April and imaged on the 11th and 25th of June. They experienced substantial environmental variation in precipitation and temperature during this period. Adaptations to temperature, precipitation, or UV exposure affect the concentrations of pigments such as anthocyanins and chlorophyll of lettuce (García‐Macías et al., 2007; Gazula et al., 2005; Marin et al., 2015; Oh et al., 2009), with the response potentially depending on the growth stage of lettuce (Becker et al., 2014). The bolting time of lettuce also depends on the climate (Al‐Said et al., 2018; Dufault et al., 2009) with differences between genotypes in the response to temperature (Dufault et al., 2009). Variation between the genotypes in climate adaptations could result in QTL that are only found in specific environments. Hence, QTLs that switch on or off over time can result from the developmental program, the response to the environment, or the interaction between these. Since in our study all accessions were grown in the same environment, we cannot disentangle the effects of the environment from that of development. In future, additional field trials under different climatic conditions are needed to show to what extent QTLs depend on the specific environment.

### Extended descriptives

In this study, we expanded the conventional trait analysis by incorporating various descriptives beyond the mean: the minimum and maximum values, standard deviation, skewness, kurtosis, and several quantiles. Mapping summary statistics describing different aspects of the trait distribution is rarely done (Evans et al., 2018) with most studies focusing only on mean traits. Some of the extended descriptives were very valuable in our study. Especially, the many quantiles proved useful to recover QTLs, we already found with mean traits with higher confidence or uncover new QTLs. This could be explained by these descriptives being less sensitive to confounders than the mean or median. For example, some of the leaves were shaded by other leaves, and this effect was not constant between accessions depending on the growth form. This effect would affect the mean color value more than the Q95. The skewness, minimum, and kurtosis showed lower heritability than the other descriptives (Figure S13) and resulted in less consistent associations (Figure S19). While the maximum worked well for the height traits, the minimum and maximum are not suitable for mapping color traits because they are very sensitive to camera artifacts and the incomplete filtering of soil pixels. Hence, we recommend quantiles over the minimum and maximum. Skewness and kurtosis might be good descriptives to find loci that affect leaf patterning, but in our population, there were not enough accessions with such phenotypes. For example, there was only one accession with clearly spotted leaves. For studies with more leaf patterning phenotypes in the population, skewness and kurtosis traits might prove more useful. The standard deviation also did not result in robust QTLs in our study. An alternative way to find QTLs related to the variance of traits is variance heterogeneity mapping approaches aimed at identifying vQTLs linked to the variance of a trait rather than the mean (Corty & Valdar, 2018; Hussain et al., 2020; Li et al., 2020; Rönnegård & Valdar, 2012). Variance heterogeneity mapping approaches use more advanced statistical models that directly model the variance as a parameter and are therefore more suitable when identifying vQTLs is the main goal of the study. The advantage of our approach is that it is easy to include in a standard GWAS workflow. Together, our results show that using extended descriptives, especially quantiles, can help to discover QTLs.

## CONCLUSION

In summary, we used drone phenotyping to perform GWAS on the plant color and height of lettuce in a large field experiment. We confirmed several previously known QTLs in field conditions and identified several novel QTLs. We showed that phenotyping at multiple time points can uncover growth‐stage‐dependent QTLs. Additionally, we demonstrate the advantage of using extended descriptives, such as quantiles to describe phenotypes, revealing QTLs that would be missed if we relied only on mean trait values. Our findings highlight that non‐destructive phenotyping using a drone can reveal the effect of genes and development on plant color and height traits under field conditions.

## METHODS

### Fields measurements

For this experiment, 194 *Lactuca sativa* accessions also used in Van Workum, Mehrem, et al. (2024) and Mehrem et al. (2024) (Table S1) were grown under field conditions near Maasbree in the Netherlands. Our accessions were a mix of different morphology groups: butterhead, cos, crisp, cutting, latin, oilseed, and stalk. The representatives of each morphology group shown in Figure 1 are LK061, LK147, LK087, LK183, LK108, LK198, and LK194, respectively. Each accession was grown on two plots, where each plot contained 30–40 individual plants (Figure S1). The seeds were germinated, and seedlings were grown at a commercial plant nursery on the 25th and 26th of March 2021 and planted in the field on the 27th and 28th of April 2021. Different trays were used for each replicate plot during the transplanting. The entire field was imaged on two time points: on the 11th of June (Day 78) and on the 25th of June (Day 93). This was done by a drone, the DJJ Matrice 210 RTK V2, which was equipped with a conventional RGB camera and a MSP camera, the Micasense Altum high resolution, and flew at a height of approximately 20 m. The MSP camera captures blue (475 nm, 32 nm bandwidth), green (560 nm, 27 nm bandwidth), red (668 nm, 14 nm bandwidth), red edge (717 nm, 12 nm bandwidth), and near‐infrared (NIR 842 nm, 57 nm bandwidth) light (AgEagle, 2020), further referred to as msp1, 2, 3, 4, and 5, respectively. Because some plants were removed between Day 78 and 93 for use in destructive measurements (not included in this study), we did not investigate the total surface area of the plots. The images captured by the drone were stitched together by the DroneWerkers company with GeoTiff software as well as the estimation of the height by 2.5D software using the overlap and difference in angle between the individual images (DroneWerkers, 2022). We validated the height measured by the drone by comparing it to heart length measurements from the 8th of June, 3 days before the first day of imaging, and found that this correlated well with the drone height measurements (Figure S2).

### Plot detection

Using the recorded geographic coordinates, individual plots within the field were isolated from the full image. Pixels in the image corresponding to plants were identified by a thresholding approach using the enhanced vegetation index (EVI). We used different EVI thresholds on the two imaging days, 0.25 and 0.4 respectively, due to differences in the overall brightness of the images between days. These thresholds were visually determined to represent the best tradeoff between not capturing soil pixels while still correctly identifying most plant pixels (Figure S3). The EVI more effectively identified plant pixels compared with the NDVI or simple ratio (SR), because it was better at distinguishing between the dark red/purple accessions and soil pixels covered in shadow.

### Trait extraction

After isolating the plant pixels per plot (30–40 plants), we calculated the color‐ and height trait traits. For each trait, we characterized the distribution per plot using the mean but also other descriptives, such as the trimmed means (excluding either the top and bottom 5, 10, or 40%) the median, the 5, 10, 25, 50, 75, 90, and 95% quantile, the minimum and maximum values, and the skewness and kurtosis of the distribution (Table S2). We used the RGB and MSP values directly as traits for GWAS. Additionally, we used the MSP data to construct several vegetation indices such as the NDVI, the structure insensitive pigment index (SIPI), the atmospherically resistant vegetation index (ARVI), the chlorophyll index red edge (CIred), the simple ratio (SR), the EVI, the normalized difference red edge index (NDRE), and the weighted difference vegetation index (WDVI) (Table 3). We also calculated different ratios of the RGB values, by taking $\log_{2}\frac{\text{Color}1}{\text{Color}2}$, and used these as phenotypes (Table S3). We took the logarithm of the ratios because it linearizes ratios such that deviations in the numerator or denominator have equal weight. We quantified the height by subtracting the mean elevation of the soil pixels of each plot from the height of each plant pixel to account for the unevenness of the terrain. We then averaged the height of every plant pixel to get the mean height. To calculate the final trait values, we took the mean values of the replicate plots per trait. All these traits were calculated for both imaging days. To quantify the change between days we took the ratio between both days as $\mathrm{day}‐\text{ratio}=\log_{2}\frac{\mathrm{Day}\;93}{\mathrm{Day}\;78}$. We also quantified the absolute differences (Day 93–Day 78) for each phenotype (Table S4), but these absolute differences were not used in the study due to redundancy with the day‐ratio traits. We investigated reproducibility and trait heterogeneity between the replicate plots and found that all traits were strongly correlated between the replicate plots (Figure S4).

**Table 3:** Vegetation indices used in this study

Trait	Name	Formula
ARVI	Atmospherically Resistant Vegetation Index	$\frac{\mathrm{NIR}-\mathrm{red}-\left(\mathrm{red}-\text{blue}\right)}{\mathrm{NIR}+\mathrm{red}-\left(\mathrm{red}-\text{blue}\right)}$
CIred	Chlorophyll Index Red Edge	$\frac{\mathrm{NIR}}{\text{RedEdge}}-1$
EVI	Enhanced Vegetation Index	$2.5\cdot \frac{\mathrm{NIR}-\mathrm{red}}{\mathrm{NIR}+6\cdot \mathrm{red}-7.5\cdot \text{blue}+1}$
NDRE	Normalized Difference Red Edge Index	$\frac{\mathrm{NIR}-\text{RedEdge}}{\mathrm{NIR}+\text{RedEdge}}$
NDVI	Normalized Difference Vegetation Index	$\frac{\mathrm{NIR}-\mathrm{Red}}{\mathrm{NIR}+\mathrm{Red}}$
SIPI^a^	Structure Insensitive Pigment Index	$\frac{\mathrm{NIR}-\text{blue}}{\mathrm{NIR}-\mathrm{red}}$
SR	Simple Ratio	$\frac{\mathrm{NIR}\;}{\mathrm{red}}$
WDVI	Weighted Difference Vegetation Index	$\mathrm{NIR}-a\cdot \mathrm{Red};\;a=\frac{\mathrm{NIR}\left(\text{soil}\right)\;}{\mathrm{red}\left(\text{soil}\right)}$

A more detailed version can be found in Table S3.

^a^ This index requires light at 800, 680, and 445 nm. Since we do not have those exact wavelengths available, we use NIR, red, and blue as an approximation.

### Phenotype clustering

Many of the traits we quantify are highly correlated. We used K‐means clustering with *K* = 10 to cluster the traits based on the absolute correlation matrix with the kmeans() function from base R (R Core Team, 2023). We visualize these clusters as a network using the R package GGnetwork (Briatte, 2023). In this network, all traits with an absolute correlation above 0.8 are connected.

### Variant calling

To genotype all accessions in our study, we used raw Illumina short read sequences mapped against the Salinas V8 Lettuce reference genome (https://www.ncbi.nlm.nih.gov/genome/352) by Van Workum, Mehrem, et al. (2024) (European Nucleotide Archive [ENA] at EMBL‐EBI, PRJEB63589).

The BAM files generated in Van Workum, Mehrem, et al. (2024) were processed by adding read group information and marking duplicate alignments to enable using the GATK toolkit (version 4.1.3.0; Poplin et al., 2018). The processed BAM files were converted to .cram‐format for long‐term storage using the following command for each accession:

samtools view ‐‐threads 8 ‐O cram embed_ref ‐T reference.fasta ‐C ‐o LK.cram LK.bam

We used the processed bam files as input for the variant calling with GATK HaplotypeCaller. The resulting GVCF files were split into smaller genomic chunks. All samples were then combined for each chunk using the GATK CombineGVCF tool. Next, the combined per chunk GVCFs were genotyped by GATK GenotypeVCF (Box 1). After that, variant selection and filtration of SNPs and INDELs were performed. We filtered the SNPs on Quality Depth (QD), Fisher Strand Bias (FS), Mapping Quality (MQ), Strand Odds Ratio (SOR), Mapping Quality Rank Sum Test (MQRankSum) and Read Position Rank Sum Test (ReadPosRankSum) (see Box 1 for parameters).

Box 1 **Bash code for variant calling and filtering variants**

HaplotypeCaller ‐‐params.haplotypecaller.optional = ‘‐ERC GVCF’

VariantFiltration ‘SNP’ ‐> “‐‐filter‐expression ‘QD < 2.0 || FS > 60.0 || MQ < 40.0 || SOR > 3.0 ||

MQRankSum < ‐12.5 || ReadPosRankSum < ‐8.0’ ‐‐filter‐name ‘snp_filter’ ‐‐genotype‐filter‐expression ‘DP < 2 || DP > 50’ ‐‐genotype‐filter‐name ‘dp_fail’”

‘INDEL’ ‐> “‐‐filter‐expression ‘QD < 2.0 || FS > 200.0 || SOR > 10.0 || MQRankSum < ‐12.5 ||

ReadPosRankSum < ‐8.0’ ‐‐filter‐name ‘indel_filter’”

SelectVariants ‘SNP’ ‐> ‘‐‐select‐type SNP ‐‐select‐type NO_VARIATION’

‘INDEL’ ‐> ‘‐‐select‐type INDEL ‐‐select‐type MIXED’

Lastly, all the separate chunks were merged into one VCF file.

Next, we filtered the SNPs based on their distributions. SNPs were excluded when they had less than 10 of either reference alleles (REF) or alternative alleles (ALT), leaving 12 976 955 SNPs. The threshold of at least 10 accessions with either allele corresponds to a minor allele frequency (MAF) of ~5%. Next, we clumped the SNPs based on linkage and distribution to avoid redundant test and reduce computation (Prive et al., 2018).

The clumping was done as follows: Starting at the first variant in the list of all SNPs, we clumped each SNP in the next 2000 SNPs that differed from the initial variant in less than five accessions (out of 198 accessions, 194 of which were phenotyped in this study). The clump was then removed from the list and stored for later use. This process was iteratively repeated until all SNPs were clumped. The positions of each SNP within a clump were recorded and can be traced using the SNP metadata. The allele distribution of the SNP with the highest MAF was taken as the representative of each clump. After clumping, 2 491 009 clumped SNPs were left. Since all accessions were of single seed descent, we filtered SNPs with more than eight heterozygous accessions or less than eight of the reference or alternative alleles, leaving 1 154 639 SNPs. The matrix with these SNPs coded as 0 for reference allele, 1 for heterozygous, and 2 for alternative allele was used for GWAS.

We calculated the linkage disequilibrium in our population with the LD.decay() function from the sommer package (Covarrubias‐Pazaran, 2016).

### GWAS

GWAS was performed on all traits using R (version 4.3.1) (R Core Team, 2023) and the Ime4QTL package (Ziyatdinov et al., 2018) as in Mehrem et al. (2024). The SNP covariance matrix (R‐function cov(), Table S5) was used to account for population structure. Taking a conservative approach to multiple testing correction, we only considered SNPs with $-\log_{10}\left(P\right)\;\text{value}>7$ significantly linked to a trait. All significant SNPs found with GWAS for all individual traits are available in Table S6. We used the gene annotation from Van Workum, de Ridder, et al. (2024) to look for candidate genes in the discovered QTLs. To rule out that the clumping procedure had a major effect on the GWAS results, we also ran the GWAS with the unclumped SNPs for five example traits (12 976 955 SNPs, Figure S5) and compared it with the results with the clumped genetic map (1 154 639 SNPs, Figure S6). These results showed a similar QTL profile with the clumped SNPs and can be used to investigate the QTLs reported below with increased resolution. All SNPs with –log_10_(*P*) > 5 for these traits are available in Table S7. To investigate the robustness of the two replicate plots per accession, we performed the GWAS on all traits for both replicates separately. The QTL profile was very similar between replicate 1, replicate 2 and the mean of the replicates (Figure S7), showing that heterogeneity between the replicates was not a major issue in this experiment.

### Heritability

Broad‐sense heritability (BSH) for each trait was calculated by taking the ratio of the between genotype variance and the total variance, using the mean square values obtained using anova as measures of variance. To verify this anova‐based approach, we also calculated the heritability using a more established approach based on quantifying the genotypic and residual variance using the lme4 package as in Maulana et al. (2022): REML <‐ lmer(trait ~1 + (1|accession)), followed by extracting the genotypic (Vg) and residual (Ve) variance components and calculating the BSH as Vg/(Vg + Ve). This approach gave identical BSH values to our anova‐based approach (Figure S8).

### Robust loci and Figure 5

To visualize and report QTLs as one or a few points in Figure 5 and Table 2, we binned the genome into ~1 Mbp bins and collected the SNP with the lowest *P*‐value per bin (Tables S8 and S9). We also used the binned *P*‐values to define “robust loci” where multiple traits map to the same genomic region as regions where three traits in a 2.5 Mbp window had a significant $\left[{-\log}_{10}\left(P\right)\;\text{value}>7\right]$ association. The lower and upper boundaries of robust loci was determined by searching for the most distant bin with –log_10_(*P*‐value) ≥ 7 in a 20 Mbp window around the bin with the lowest *P*‐value. Redoing the analysis with extended descriptives required stricter criteria to define robust loci due to the larger number of traits. In this case, we defined robust loci as regions where at least five unique traits in a 2.5 Mbp window had an association with –log_10_(*P*‐value) ≥ 9 and set the boundaries as the distant most SNPs in a 20 Mbp window with a –log_10_(*P*‐value) > 9. We used rectangles to highlight robust QTLs in Figure 5 and Figure S9.

## AUTHOR CONTRIBUTIONS

RO, MP, GvdA, and BLS conceived the study. KS, RO, MP, GvdA, SLM, JvL, EvdB, and SK performed the experiment. ALvE, RD, RO, SLM, and BLS extracted the data. ALvE, RD, and BLS performed the investigation. RD, ALvE, and BLS wrote the manuscript with input from all co‐authors.

## CONFLICT OF INTEREST

The authors declare that they have no conflicting interests.

## Supporting information


**Figure S1.** Overview of the field layout.
**Figure S2.** Relationship between drone height measurements and manual heart length measurements.
**Figure S3.** Plant pixel selection using EVI threshold for five accessions.
**Figure S4.** Comparison of traits from both replicates.
**Figure S5.** Manhattan plots of GWAS with the unclumped SNP matrix for five example traits.
**Figure S6.** Manhattan plots of GWAS with the clumped SNP matrix for five example traits.
**Figure S7.** Comparison of GWAS results for both replicates and mean of replicates.
**Figure S8.** Comparison of Broad‐sense heritability calculated by anova and by the lme4 package.
**Figure S9.** Comparison of using only the mean traits or using many extended descriptives.
**Figure S10.** Comparison between plants with low and high log_2_(green/blue) ratio for day 78.
**Figure S11.** Comparison between plants with low relative red and high relative red on day 78.
**Figure S12.** Broad‐sense heritability of all phenotypes.
**Figure S13.** Broad‐sense heritability of all descriptives.
**Figure S14.** Clustering for different thresholds.
**Figure S15.** The linkage disequilibrium in *L. sativa*.
**Figure S16.** Comparison of height on both days and the day‐ratio.
**Figure S17.** The traits causing the QTL on chromosome 8.
**Figure S18.** Details about the clustering on all traits, including extended descriptives.
**Figure S19.** The QTLs found per extended descriptive.

**Table S1.** LK_lines. Information about all the lettuce lines.
**Table S2.** Statistical_definitions. Description of all used summary statistics.
**Table S3.** Pheno_definitions. Description of all used phenotypes.
**Table S4.** Phenotypes. All phenotypes in long format.
**Table S5.** Kinship. Kinship matrix used to correct for population structure during GWAS.
**Table S6.** All significant SNPs.
**Table S7.** Full SNPs 5 traits.
**Table S8.** QTLs. Prominent loci found when using only the mean traits.
**Table S9.** QTLs. All prominent loci found when using the extended descriptives.
**Table S10.** Heritability scores of all traits.
**Table S11.** Correlation matrix. Correlation of all traits with each other from 0 to 1.
**Table S12.** LD_decay. Linkage disequillibrium decay.
**Table S13.** *P* values of all significant (−log_10_ >7) SNPs after GWAS of all phenotypes.
**Table S14.** Height.mean.day2. GWAS result on height for generating Figure 4.
**Table S15.** gbrat.mean.day2. GWAS result on green blue ratio for generating Figure 4.
**Table S16.** Rededge.mean.day2. GWAS result on red.edge for generating Figure 4.
**Table S17.** Height.traits. All height traits in one sheet.

## Data Availability

The code for the variant calling pipeline using GATK is available on UMCUGenetics/NF‐IAP at bqsr_optional_gatk_parameterize. The scripts for making the SNP map from the filtered VCF file and for the image processing, GWAS, and figures in this manuscript are available on https://github.com/SnoekLab/Dijkhuizen_etal_2025_Drone. Data available at https://doi.org/10.24416/UU01‐S5FCM9. This includes all raw data, all intermittent steps, the data required to generate all figures, and data on the weather during the experiment.
