# Supplementary material for: From aerial drone to quantitative trait locus: leveraging next‐generation phenotyping to reveal the genetics of color and height in field‐grown Lactuca sativa
Source: Plant J. 2025 Aug 13;123(3):e70405. doi: 10.1111/tpj.70405 (PMC12349928; doi:10.1111/tpj.70405)
Supplement: Supplementary file 1 — Figure S1. Overview of the field layout. Figure S2. Relationship between drone height measurements and manual heart length measurements. Figure S3. Plant pixel selection using EVI threshold for five accessions. Figure S4. Comparison of traits from both replicates. Figure S5. Manhattan plots of GWAS with the unclumped SNP matrix for five example traits. Figure S6. Manhattan plots of GWAS with the clumped SNP matrix for five example traits. Figure S7. Comparison of GWAS results for both replicates and mean of replicates. Figure S8. Comparison of Broad‐sense heritability calculated by anova and by the lme4 package. Figure S9. Comparison of using only the mean traits or using many extended descriptives. Figure S10. Comparison between plants with low and high log2(green/blue) ratio for day 78. Figure S11. Comparison between plants with low relative red and high relative red on day 78. Figure S12. Broad‐sense heritability of all phenotypes. Figure S13. Broad‐sense heritability of all descriptives. Figure S14. Clustering for different thresholds. Figure S15. The linkage disequilibrium in L. sativa. Figure S16. Comparison of height on both days and the day‐ratio. Figure S17. The traits causing the QTL on chromosome 8. Figure S18. Details about the clustering on all traits, including extended descriptives. Figure S19. The QTLs found per extended descriptive. [file TPJ-123-0-s001.docx]

**Supplementary figures**


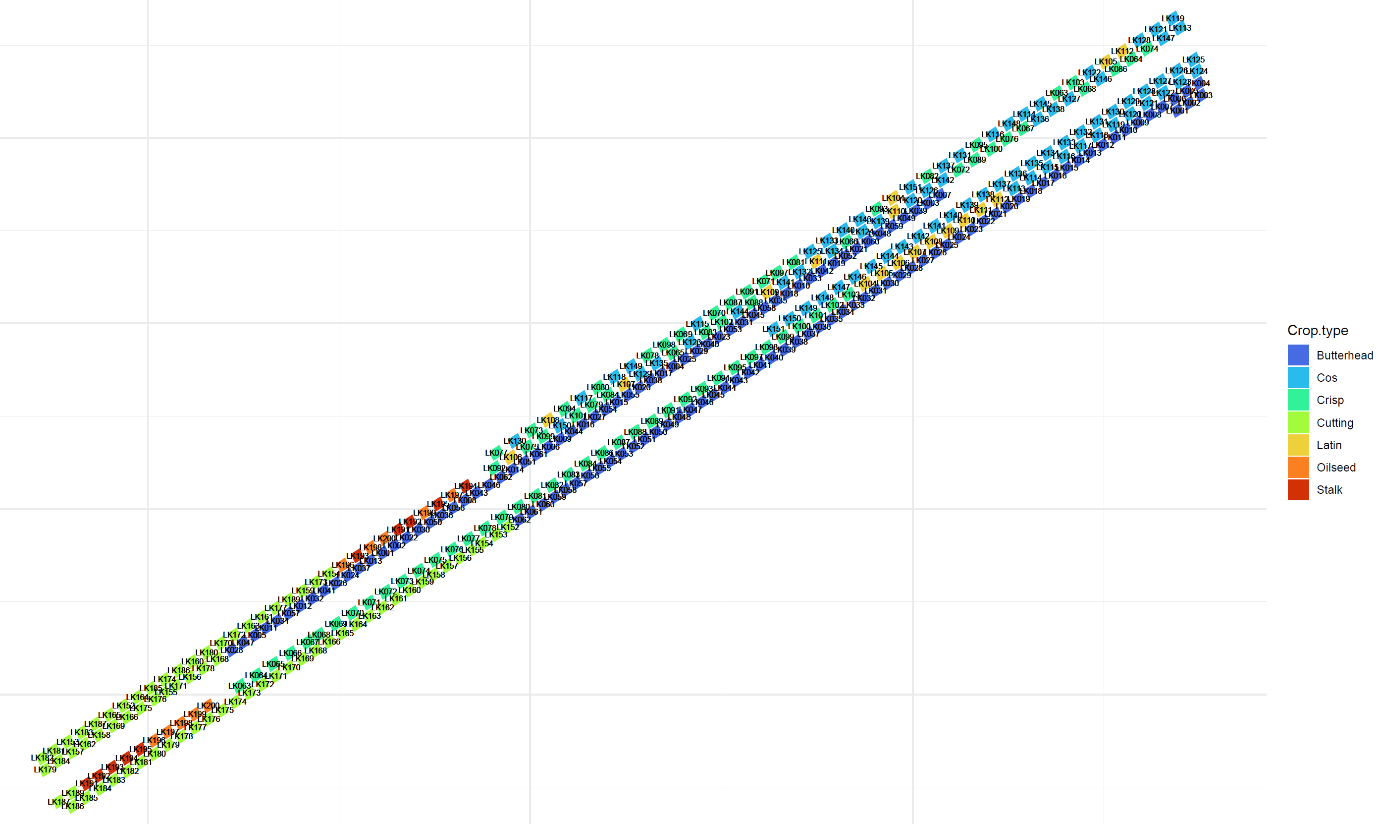


**Supplementary Figure 1: Overview of the field layout.** Positions of the plots are shown in color and labelled by genotype id. The different colors indicate the different horticultural (or crop) types. Upper diagonal contains replicate 1 plots and lower diagonal contains replicate 2 plots.


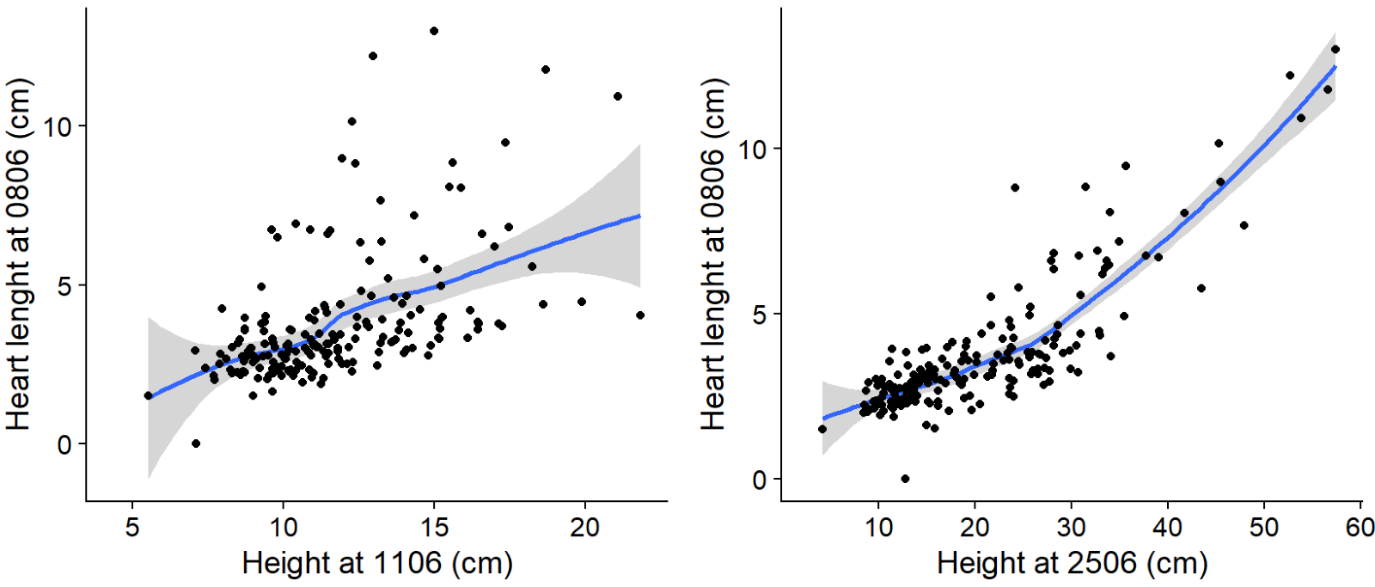
**Supplementary Figure 2: Relationship between drone height measurements and manual heart length measurements.** Relationship between height measured by drone on **A)** the 11^th^ of June and **B)** the 25^th^ of June and the heart length measured manually on the 8^th^ of June.


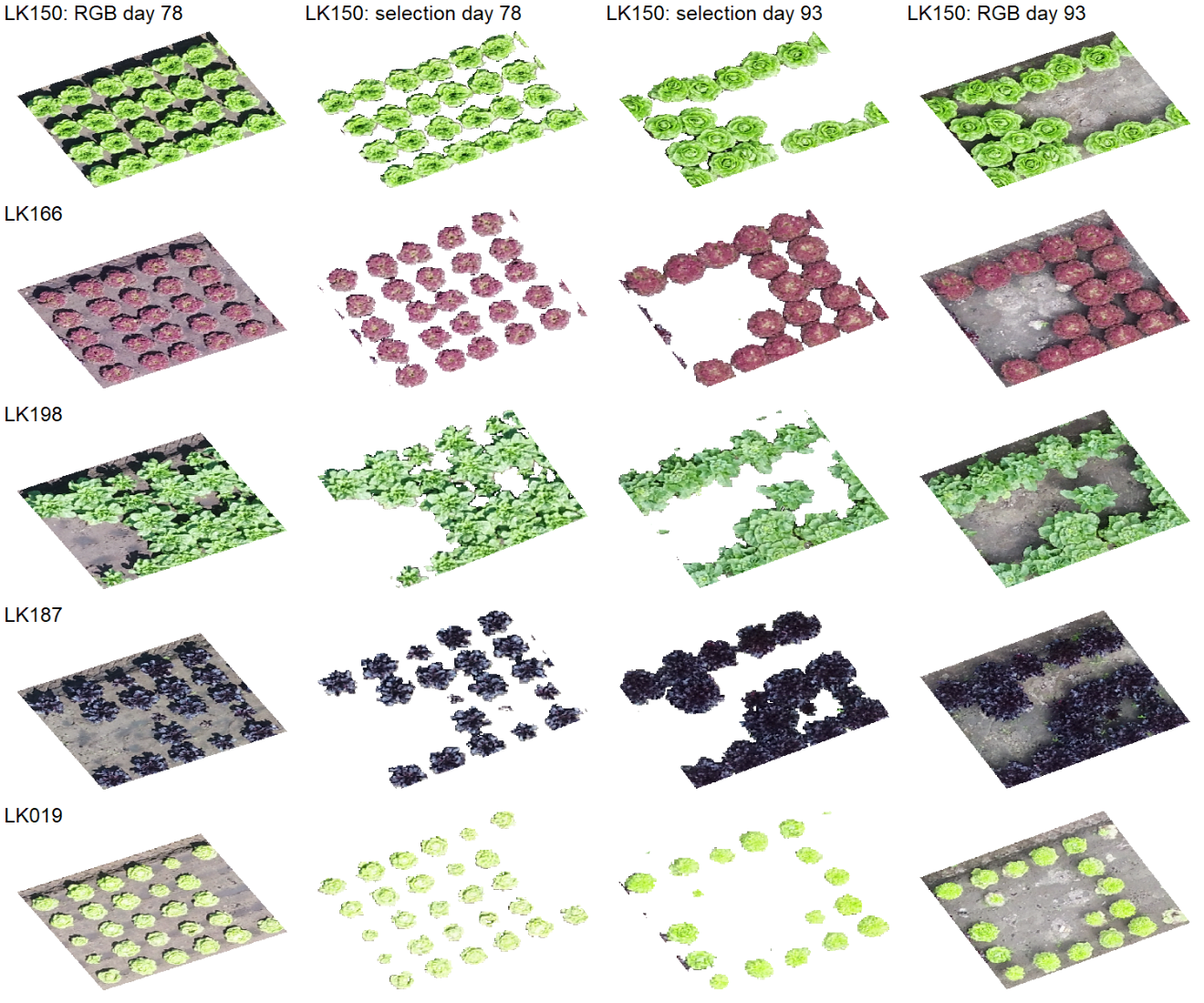


**Supplementary Figure 3: Plant pixel selection using EVI threshold for five accessions.** The first column shows the RGB image for the accessions on day-78. The second column shows the plant pixels after thresholding on day-78 with an EVI threshold of 0.25. The third column shows a similar thresholding for day-93 with a threshold of 0.4.


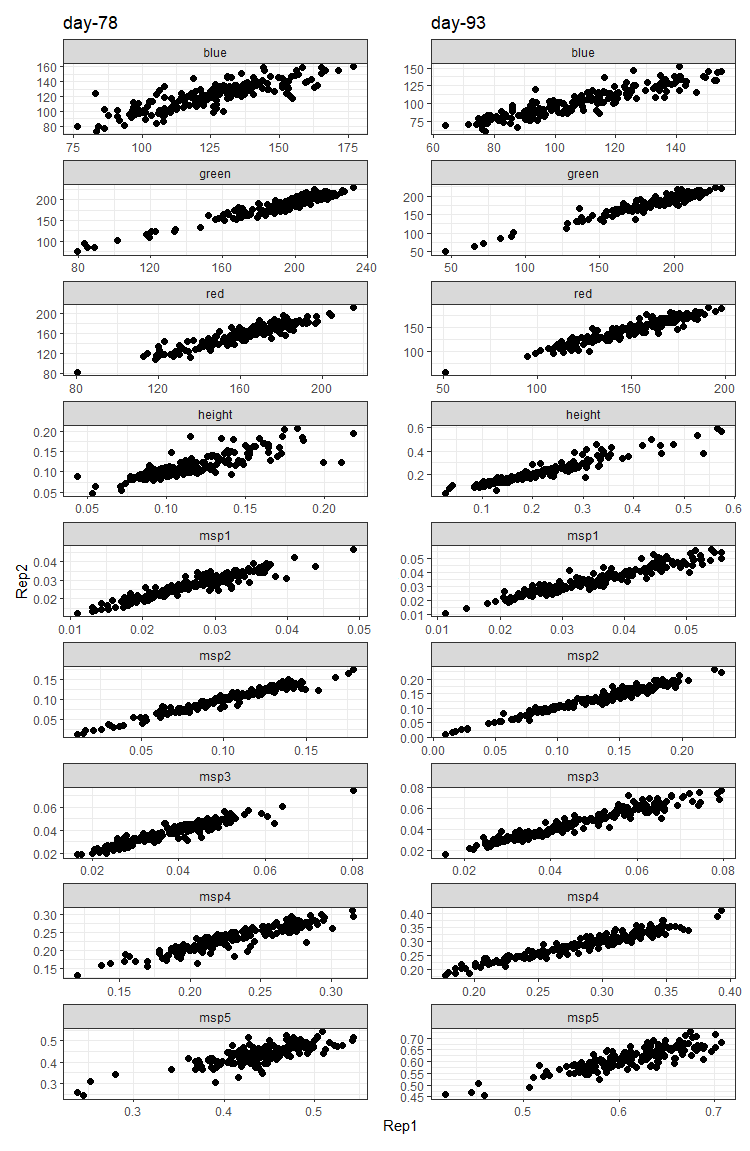
**Supplementary Figure 4: Comparison of traits from both replicates.** Comparison of RGB and multispectral traits between replicate 1 and 2 on day-78 and day-93.


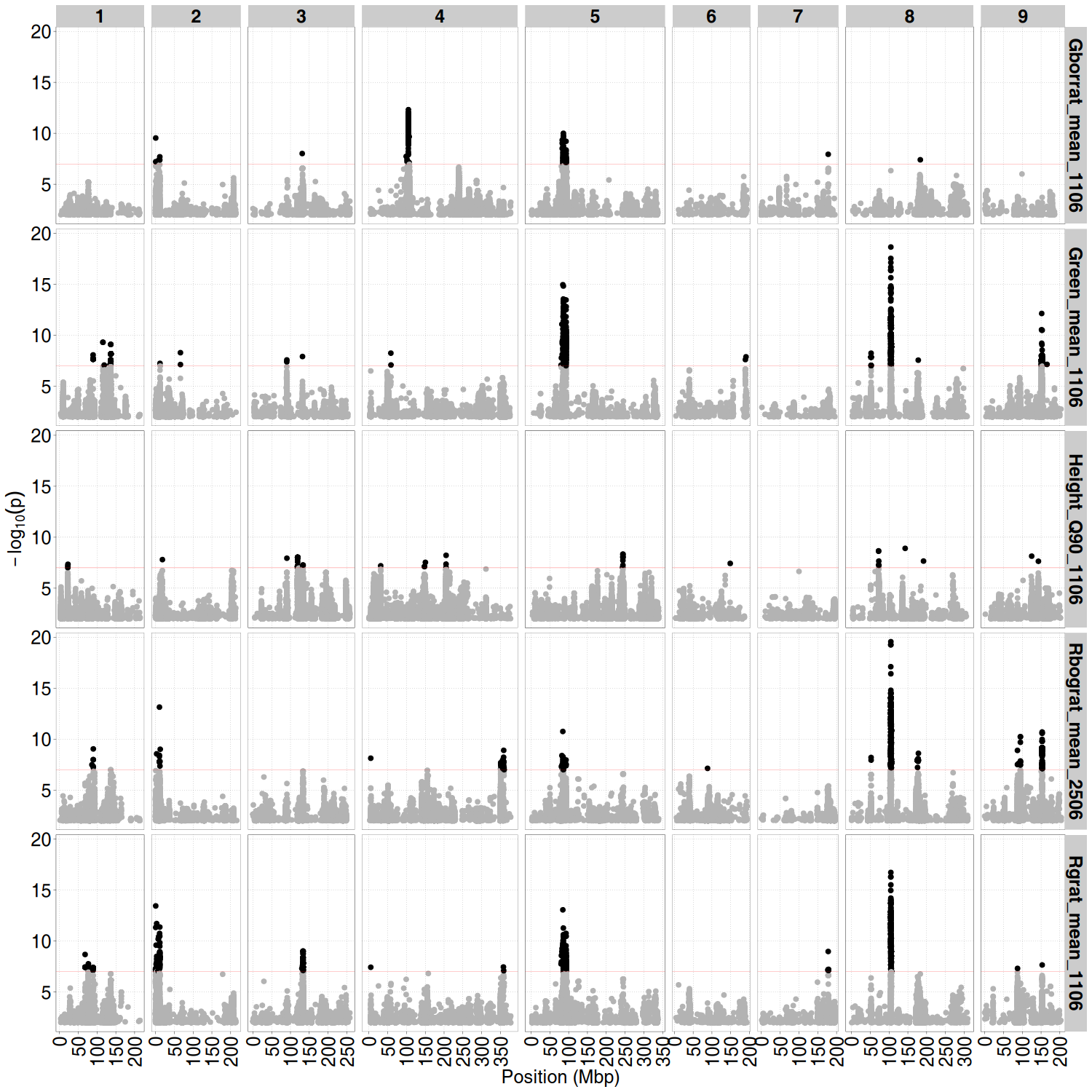
**Supplementary Figure 5: Manhattan plots of GWAS with the unclumped SNP matrix for five example traits.** 12,976,955 quality- and MAF filtered but unclumped SNPs were tested. All SNPs with log10(P) > 5 are in **Supplementary Table 7**.


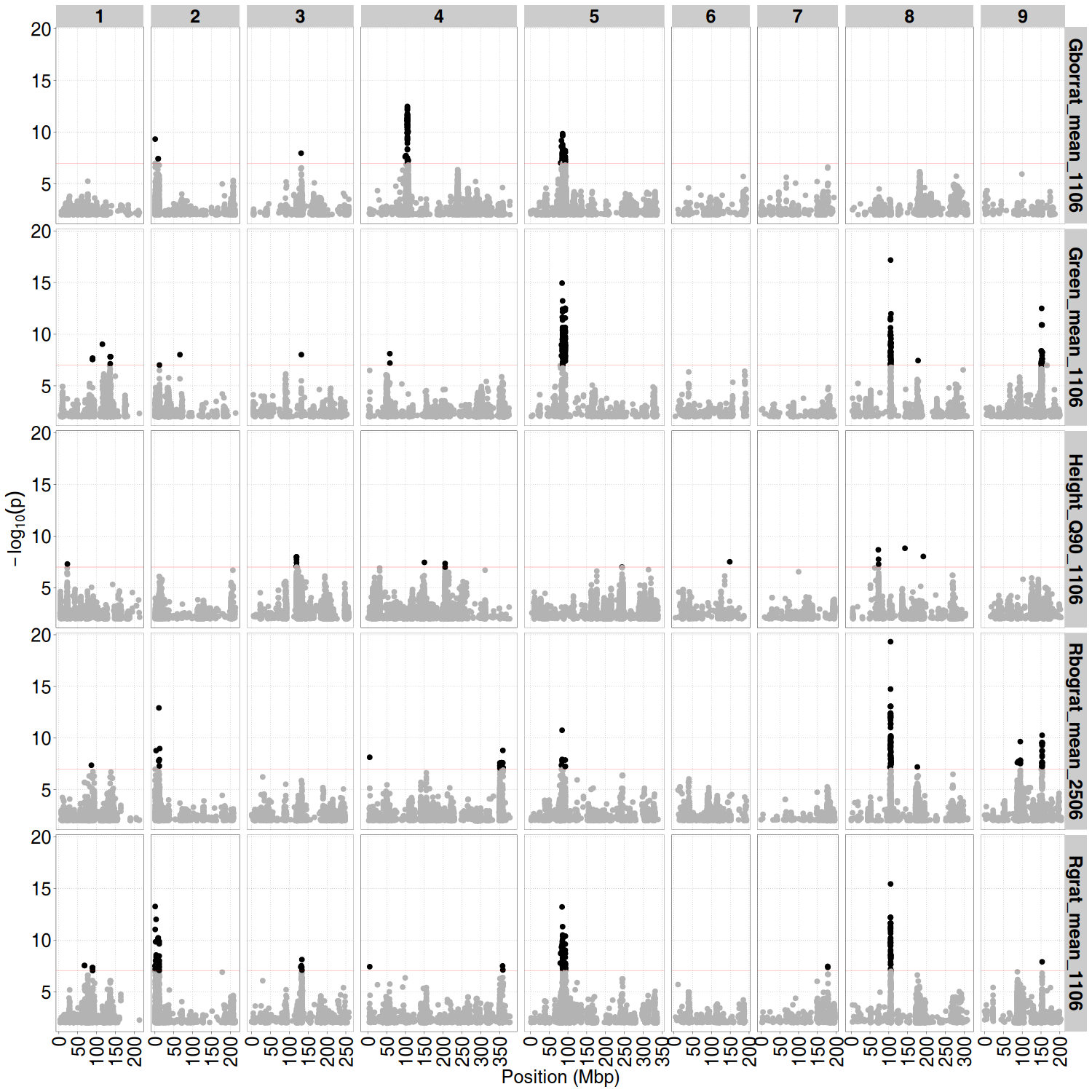
**Supplementary Figure 6: Manhattan plots of GWAS with the clumped SNP matrix for five example traits.** 1,154,639 quality filtered, MAF filtered, and clumped SNPs were tested.

**
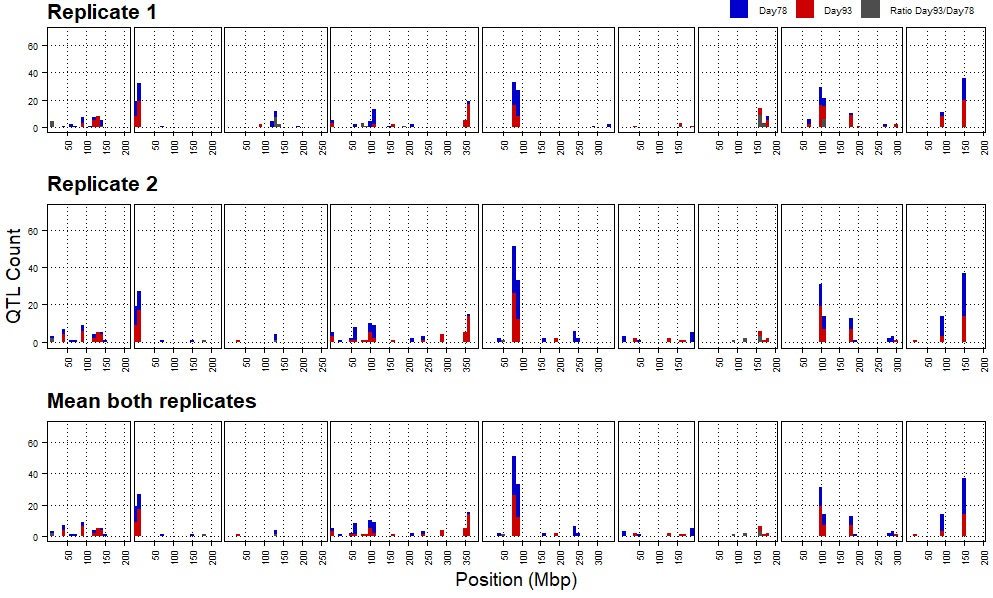
**

**Supplementary Figure 7: Comparison of GWAS results for both replicates and mean of replicates.** Histogram per replicate and mean showing all significant (-log_10_(pvalue) > 7) SNPs.


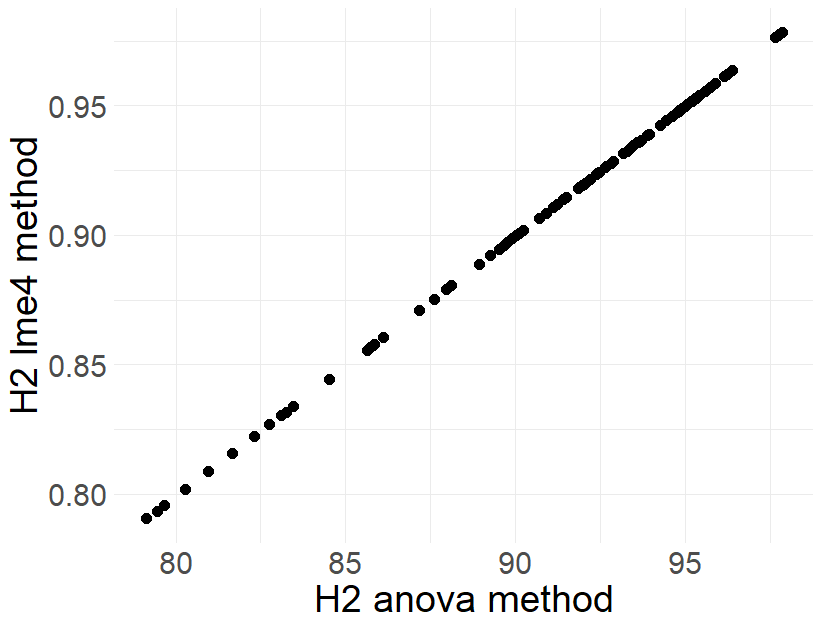


**Supplementary Figure 8: Comparison of Broad-sense heritability calculated by anova and by the lme4 package.** Broad-sense heritability was calculated with the lme4 package and with anova. For the anova approach we calculated the ratio of between genotype variance and total variance, using the mean square values obtained by anova as a measure of variance. Both methods results in the exact same values.

**
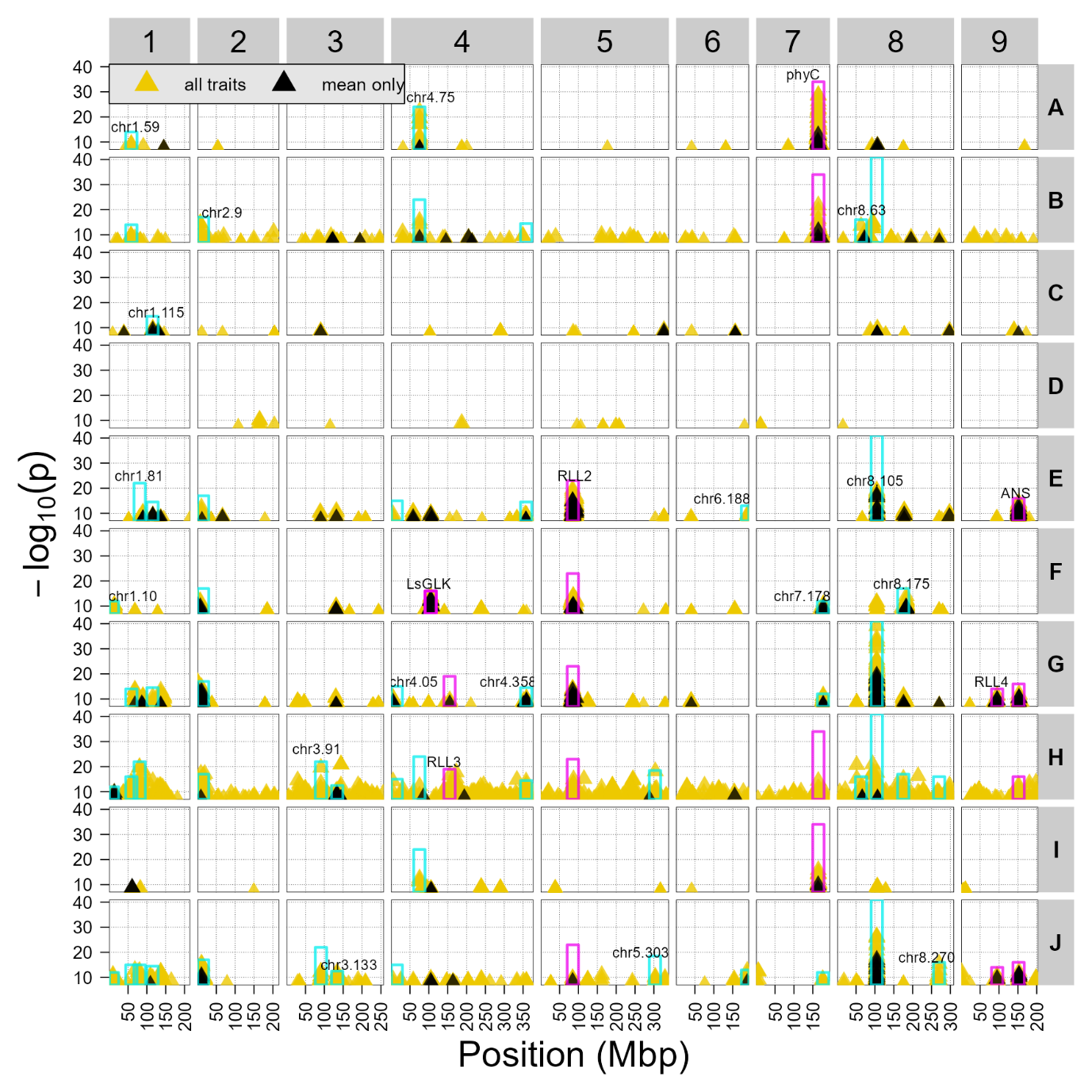
Supplementary Figure 9: Comparison of using only the mean traits or using many extended descriptives.** Every row represents a cluster of traits. The black dots represent a mean trait, while the yellow dots represent extended descriptives. QTLs that have been previously reported are highlighted with a purple rectangle while (to our knowledge) new QTLs are shown with a teal rectangle. For overlapping loci only the most significant was highlighted.


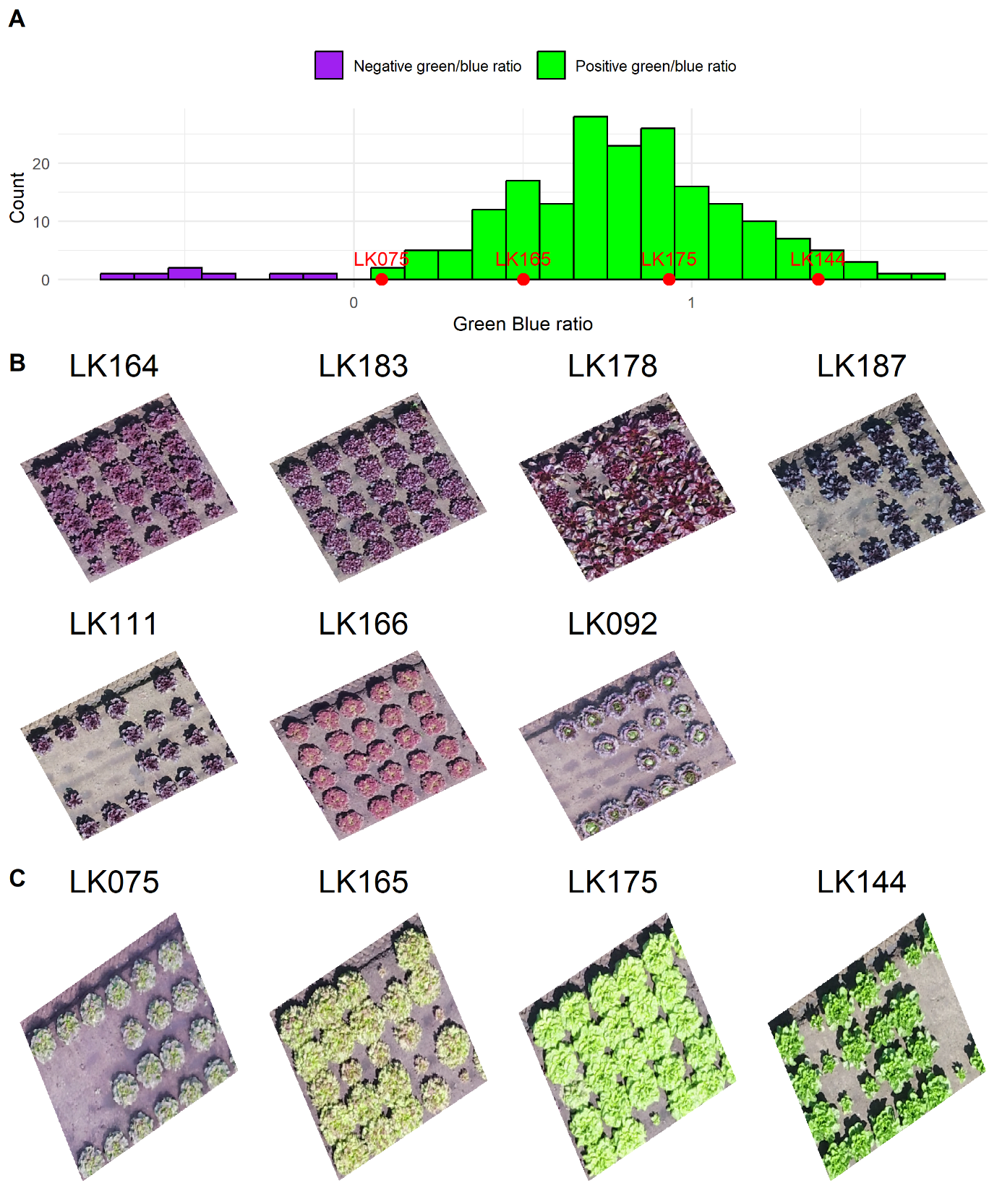


**Supplementary Figure 10:** Comparison between plants with low and high log_2_(green/blue) ratio for day-78. **A**) Histogram of all green-blue ratios. **B**) All 7 accessions with a negative green/blue ratio, ordered from lowest to highest. All 7 of these accessions show a purple color that we typically associate with anthocyanin. **C**) Four plants with a positive green/blue ratio, order from lowest to highest. The exact position of these examples is also shown in the histogram.


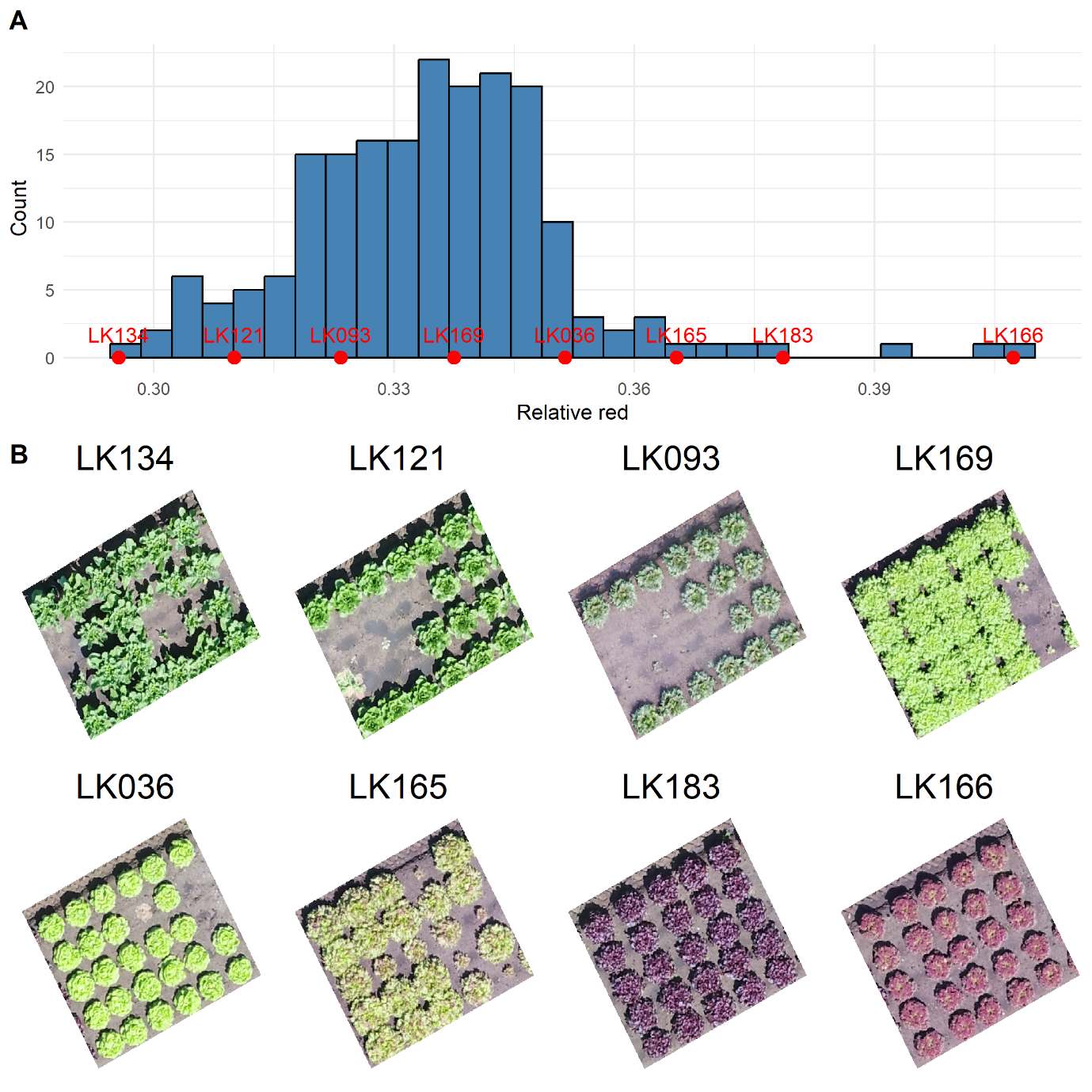


**Supplementary Figure 11:** Comparison between plants with low relative red and high relative red on day-78. **A**) Histogram of all relative red values on day-78. **B**) Examples of plants with low and high relative red. Order from low to high. The values of these accessions is annotated in the histogram.


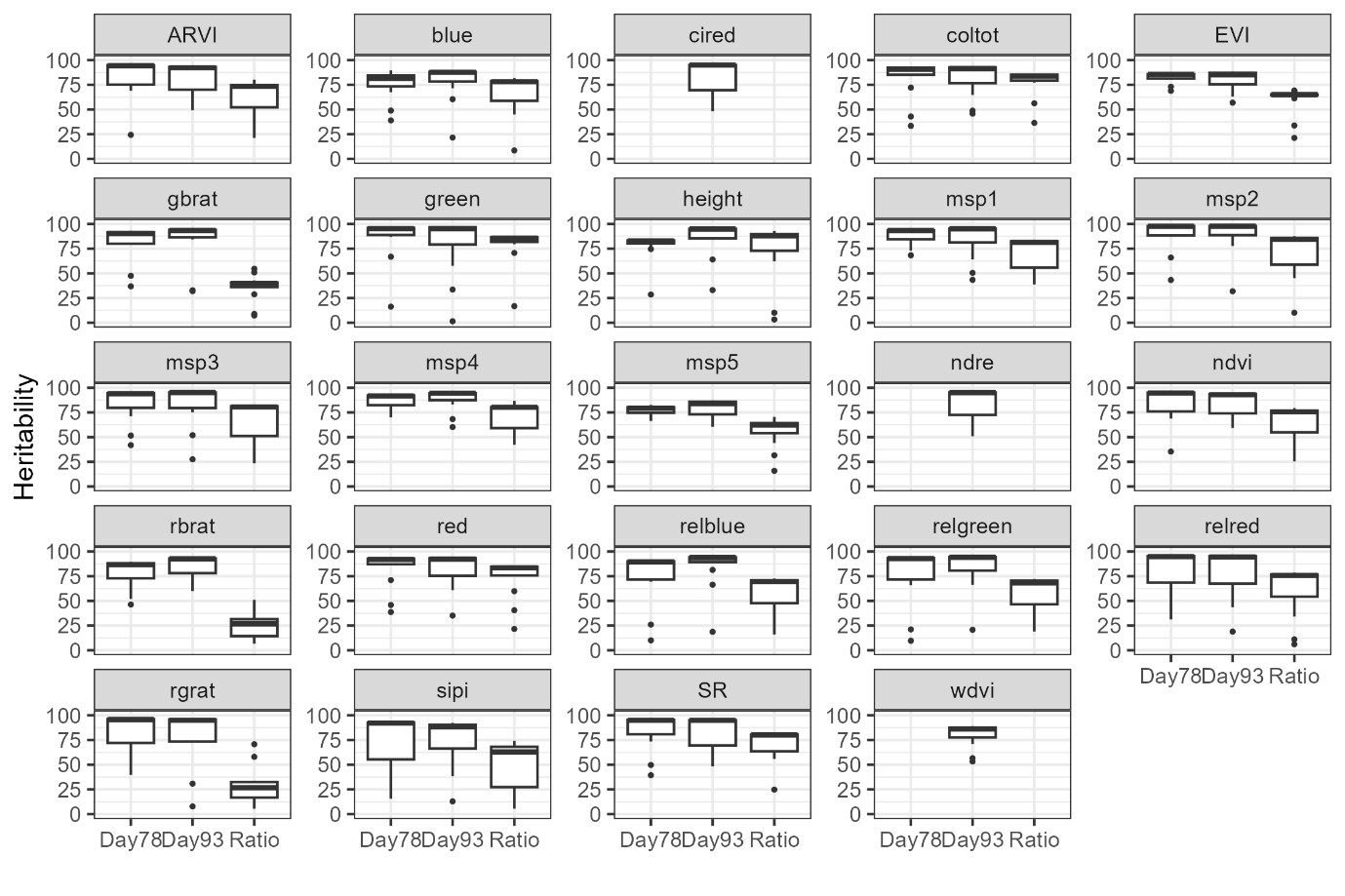
**Supplementary Figure 12: Broad-sense heritability of all phenotypes.** Heritability is shown for day-78, day-93 and the ratio of the trait between those two days for the mean and all extended descriptives. Due to an error with the drone cired, ndre and wdvi are missing for day-78.


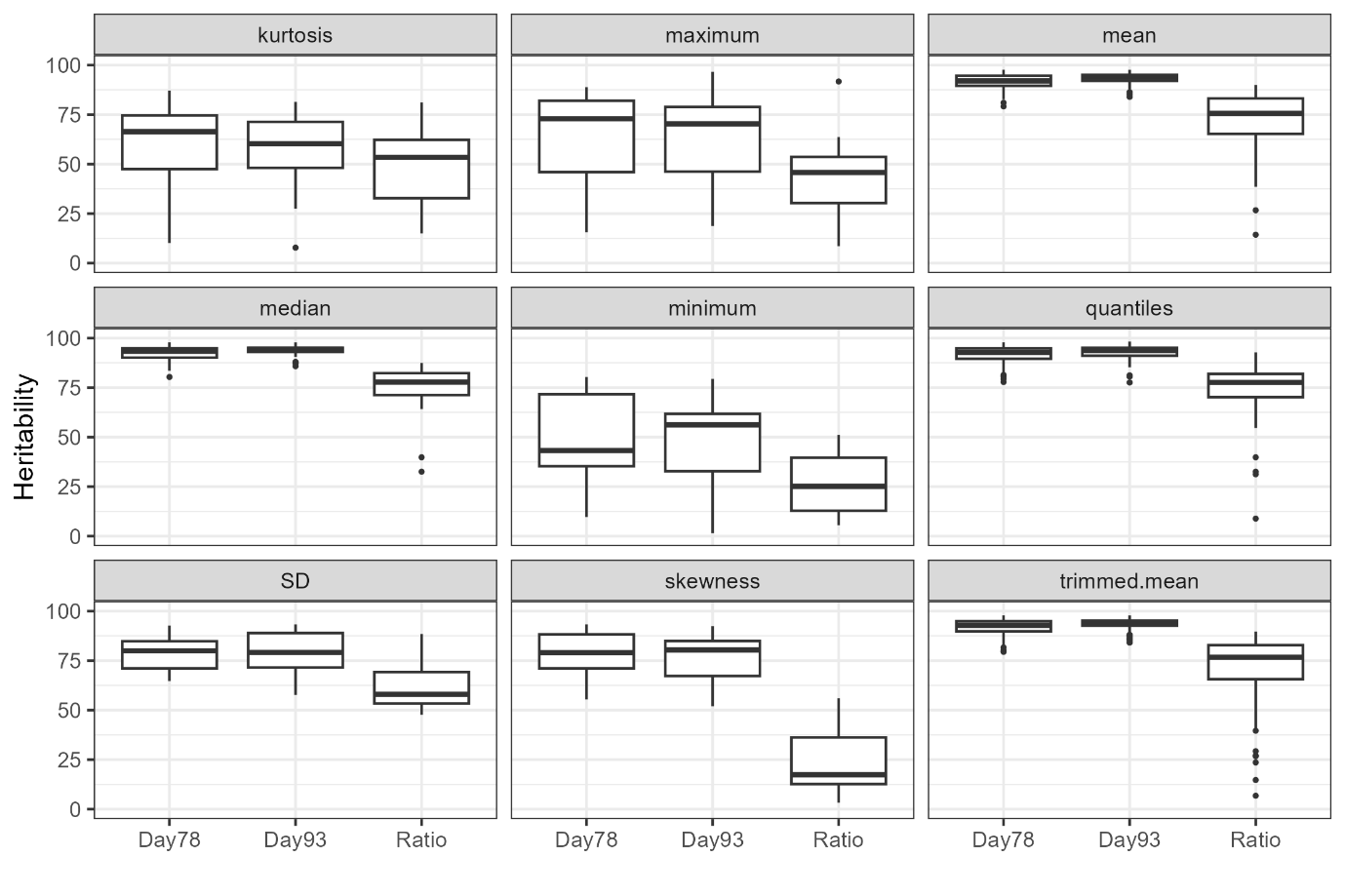


**Supplementary Figure 13: Broad-sense heritability of all descriptives.** Heritability is shown for day-78, day-93 and the ratio of the trait between those two days.


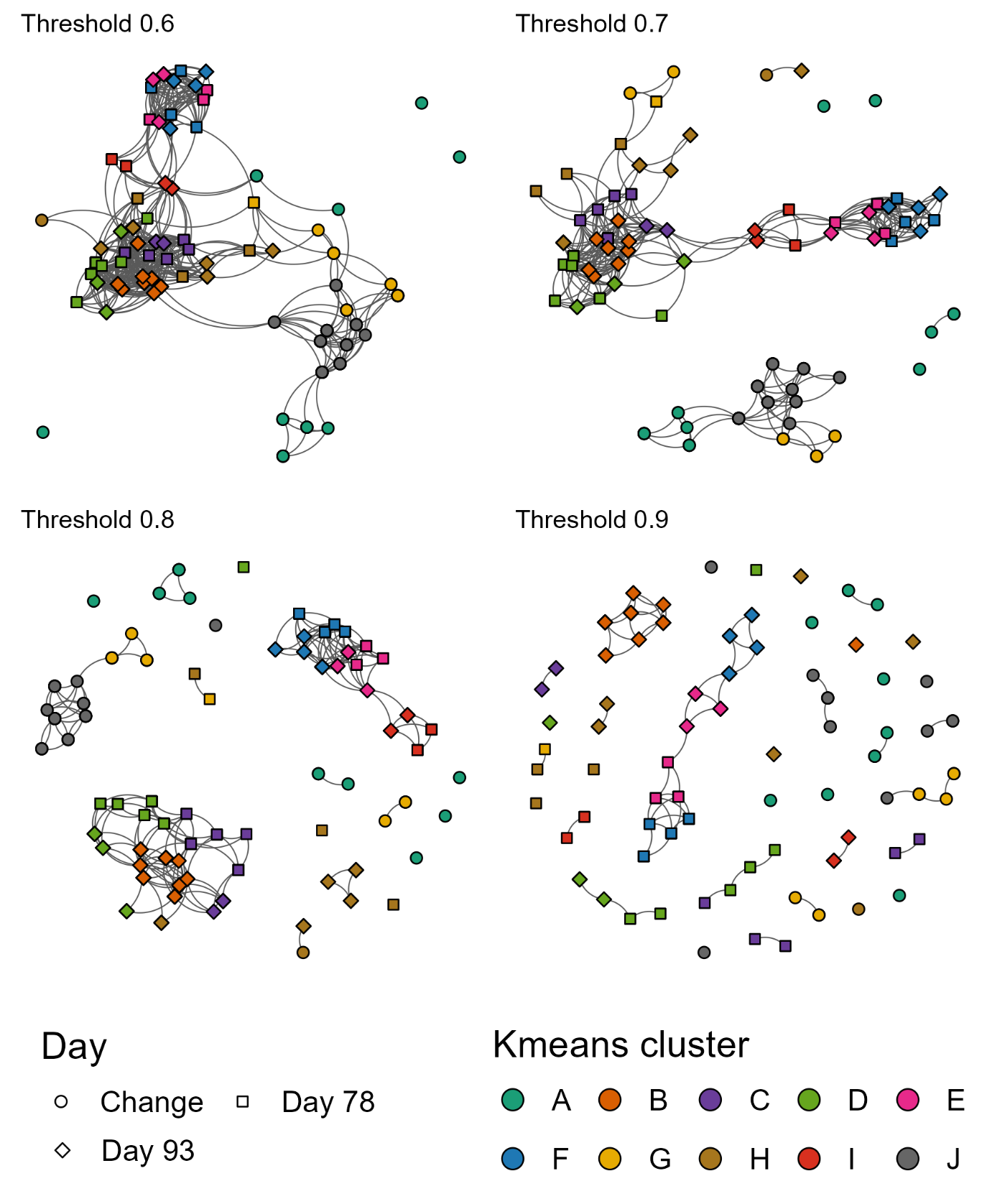
**Supplementary Figure 14: Clustering for different thresholds.** The same clustering from **Figure 3** is repeated here with 4 different thresholds. The connectiveness increases with a lower threshold.


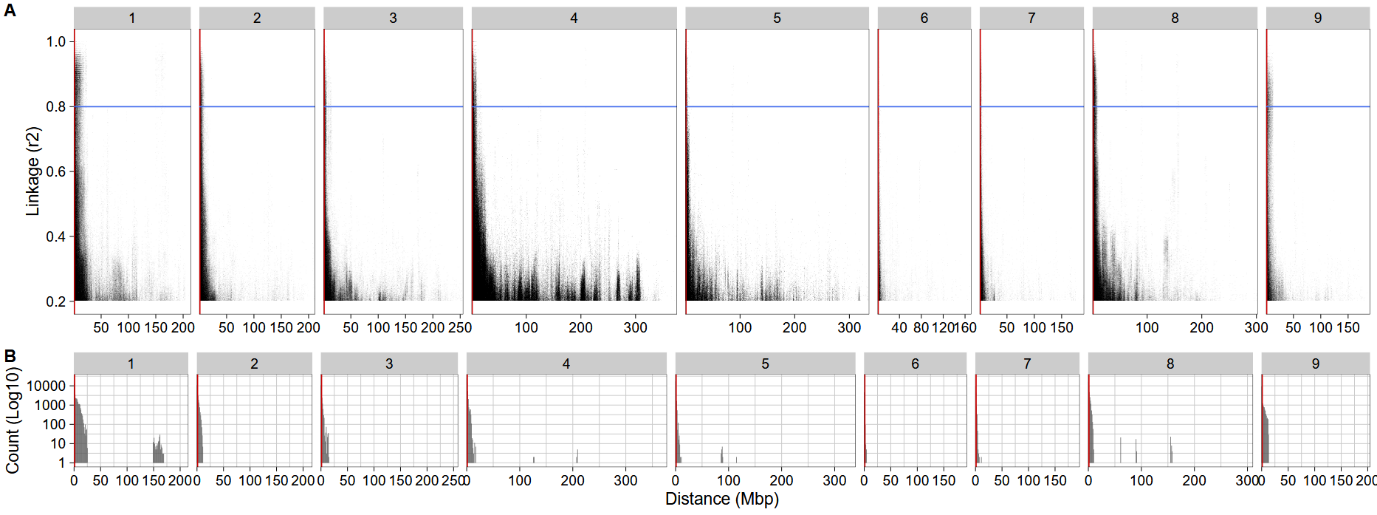


**Supplementary Figure 15: The linkage disequilibrium in *L.Sativa*.** **A**) The linkage disequilibrium for each chromosome. The blue line shows the threshold of 0.8 linkage. **B**) A histogram showing the most common linkage distance where the linkage is > 0.8.

**
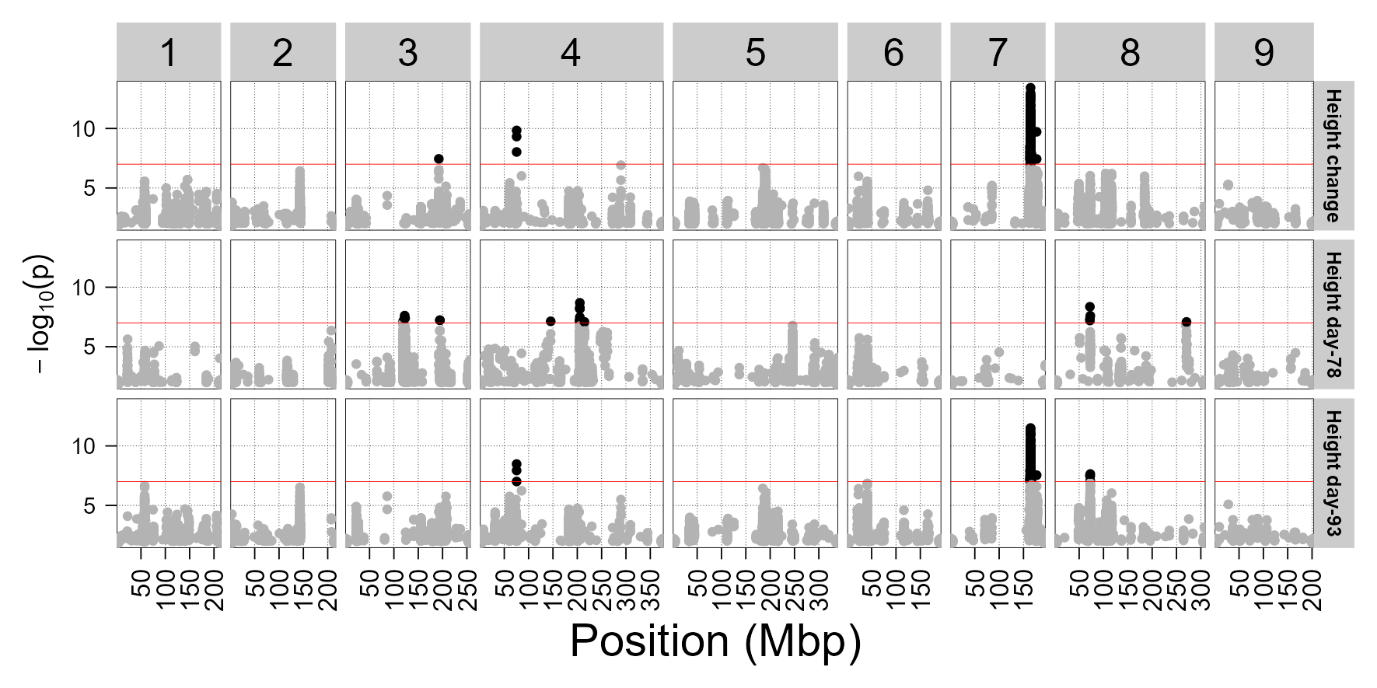
Supplementary Figure 16: Comparison of height on both days and the day-ratio.** The x-axis represents the genome of *L. sativa* in megabasepairs (Mbp). Chromosome numbers are shown on top. The y-axis represents the p-value of the GWAS test associating SNPs with trait variation. The red line at -log_10_(p-value) = 7 represents a conservative significance threshold. SNPs above this threshold are shown in black and SNPs below the threshold are shown in grey. The traits shown here are the height on day-78, the height on day-93 and the absolute change in height between those days. The QTL on chromosome 7 containing Phytochrome C is present on day-93 and the change between both days but absent on day-78.


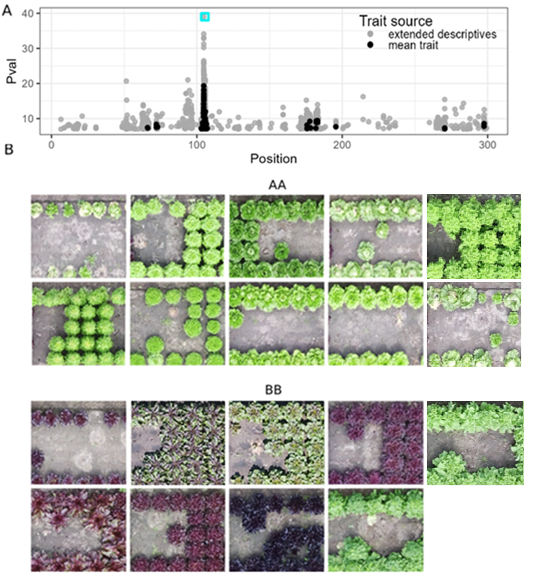
**Supplementary Figure 17:** **The traits causing the QTL on chromosome 8**. **A**) A zoom in on the combined results for all traits for chromosome 8 with the position in Mbp on the x-axis and the -log10(pvalue) on the y-axis. A highly significant SNP located at 105 Mbp is circled in teal. All mean traits are shown in black while extended descriptives are shown in grey. **B**) Accessions, on day-93, with contrasting alleles for the SNP circled in panel A. Investigating 10 random accesions (out of 180) homozygous for the reference allele we see shows green accessions. Examining all 9 accessions homozygous for the alternative allele shows that 5 out of 9 accessions have the typical purple anthocyanin color, and the other accessions are purplish or darker green.


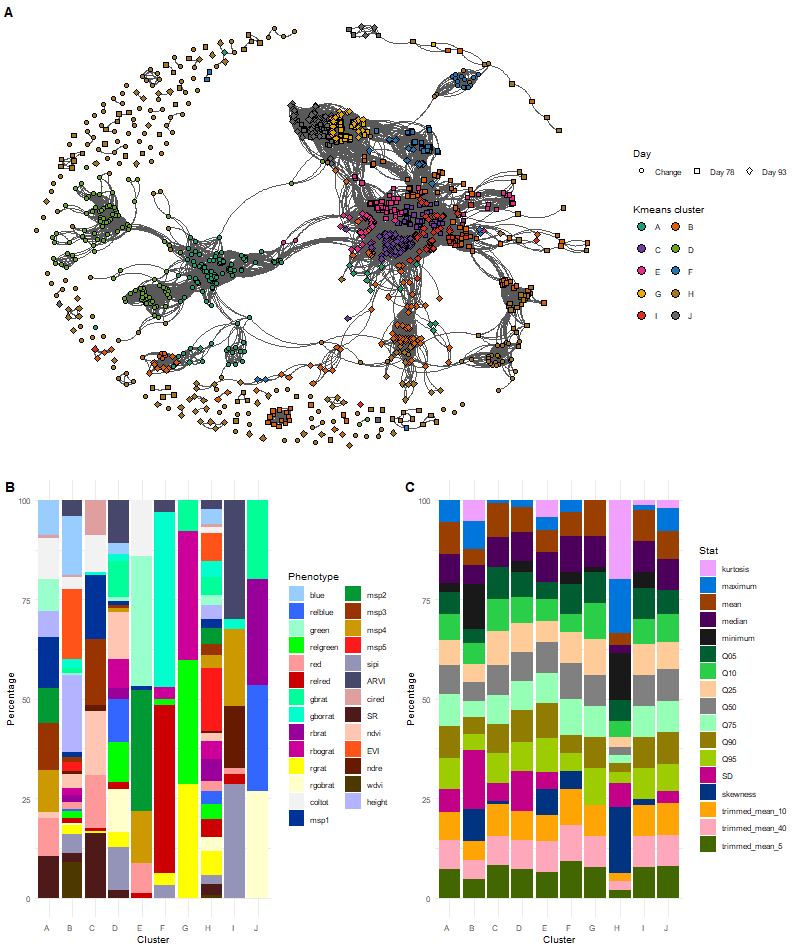


**Supplementary Figure 18:** **Details about the clustering on all traits, including extended descriptives**. Note that this k-means clustering cannot be compared to the clustering in the main text as it is not an extension but a new clustering. **A**) Network based on the trait*trait correlation matrix. Nodes represent traits, and edges represent correlations > 0.8. Color shows the cluster assignment of k-means clustering (K = 10) on the correlation matrix of all traits. **B**) Distribution of phenotypes over clusters. **C**) Distribution of descriptives over clusters.


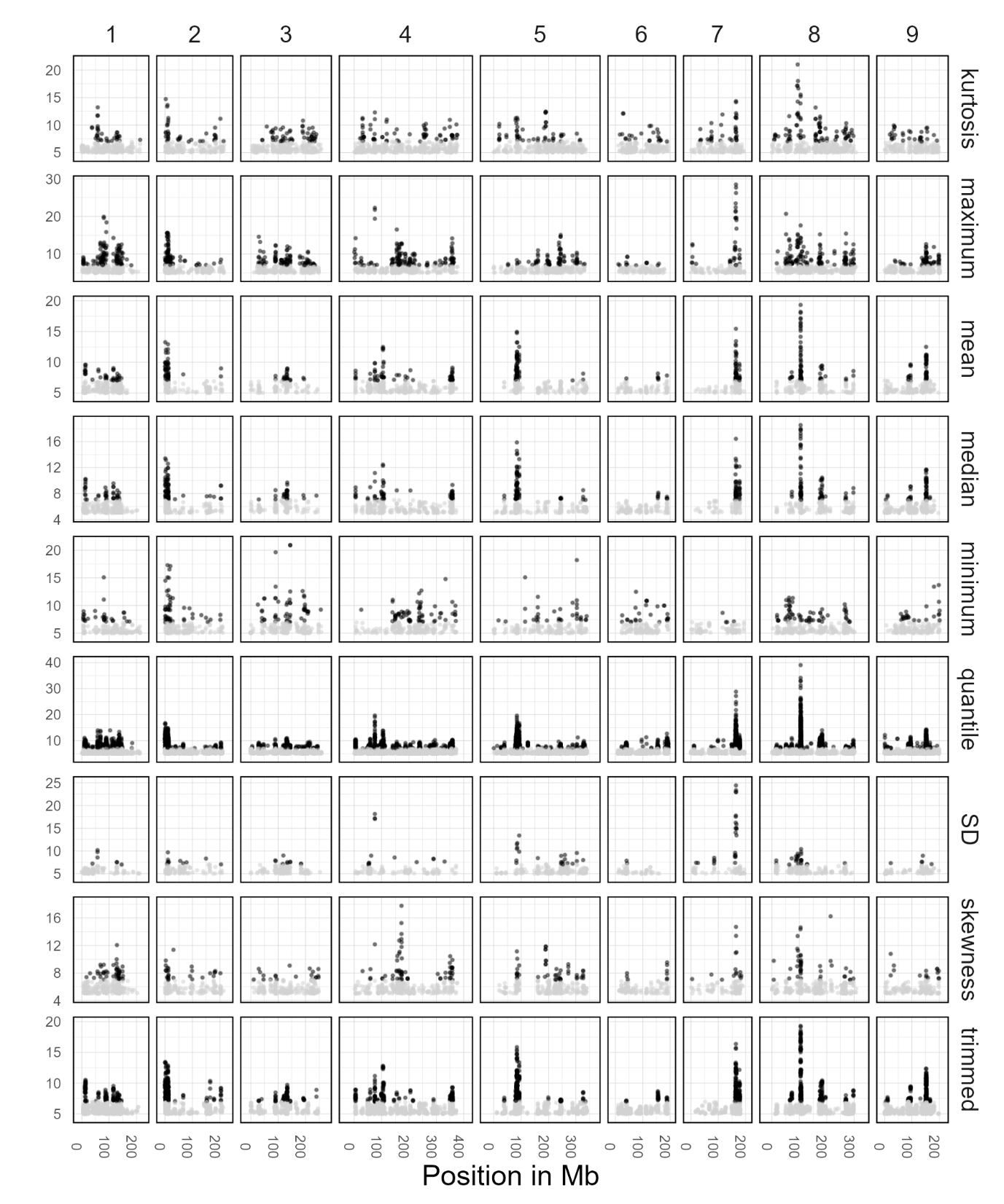
**Supplementary Figure 19:** The QTLs found per extended descriptive. Points with a -log_10_(p) > 5 but below 7 are shown in light grey.
