# Supplementary material for: From aerial drone to quantitative trait locus: leveraging next‐generation phenotyping to reveal the genetics of color and height in field‐grown Lactuca sativa
Source: Plant J. 2025 Aug 13;123(3):e70405. doi: 10.1111/tpj.70405 (PMC12349928; doi:10.1111/tpj.70405)
Supplement: Supplementary file 2 — Table S1. LK_lines. Information about all the lettuce lines. Table S2. Statistical_definitions. Description of all used summary statistics. Table S3. Pheno_definitions. Description of all used phenotypes. Table S4. Phenotypes. All phenotypes in long format. Table S5. Kinship. Kinship matrix used to correct for population structure during GWAS. Table S6. All significant SNPs. Table S7. Full SNPs 5 traits. Table S8. QTLs. Prominent loci found when using only the mean traits. Table S9. QTLs. All prominent loci found when using the extended descriptives. Table S10. Heritability scores of all traits. Table S11. Correlation matrix. Correlation of all traits with each other from 0 to 1. Table S12. LD_decay. Linkage disequillibrium decay. Table S13. P values of all significant (−log10 >7) SNPs after GWAS of all phenotypes. Table S14. Height.mean.day2. GWAS result on height for generating Figure 4. Table S15. gbrat.mean.day2. GWAS result on green blue ratio for generating Figure 4. Table S16. Rededge.mean.day2. GWAS result on red.edge for generating Figure 4. Table S17. Height.traits. All height traits in one sheet. [file TPJ-123-0-s002.zip › SupplementaryTablesCaption.docx]

**Supplementary Table 1: LK_lines. Information about all the lettuce lines.** For all lettuce accessions we show the CGN ID. CGN currently maintains one of the largest genetic resource collections of lettuce in the world. The LettuceKnowID (LKID), the morphology type, and the more common used name.

**Supplementary Table 2: Statistical_definitions. Description of all used summary statistics.** For all phenotypes we use extended descriptives. This sheet lists exactly what these extended descriptives are.

**Supplementary Table 3: Pheno_definitions. Description of all used phenotypes.** Detailed description of all phenotypes. In the code we use abbreviations for all phenotypes, this sheet explains what these abbreviations are, what these phenotypes are exactly and how they are calculated.

**Supplementary Table 4: Phenotypes. All phenotypes in long format.** Matrix containing all the phenotypes for all LKIDs. The trait and statistical description describe what trait is used and what statistic is used to measure it. The observation column shows what day the observation belongs to. 1106 is day-78, 2506 is day-93. Diff is the absolute difference and dira is the log2 fold change.

**Supplementary Table 5: Kinship. kinship matrix used to correct for population structure during GWAS.** The kinship matrix, calculated by taking the covariance matrix.

**Supplementary Table 6: All.Significant.SNPs. All significant SNPs for all traits.** All significant SNPs for all traits without any binning or clustering.

**Supplementary Table 7: FullSNPs5traits. All significant SNPs for 5 highlighted traits.** All significant SNPs with a score of -log10(pval) > 5. For 5 traits we highlight.

**Supplementary Table 8: QTLs.fig5. Prominent loci found when using only the mean traits.** All prominent loci as defined in the paper and shown in Figure 5. The phenotype column shows the phenotype with the most significant result. Phenotypes show all the phenotypes that have a significant result at the location of the most significant SNP. Position shows the position of the most significant SNP in Mbp. Pval shows the -log(pvalue). l.bound and u.bound show the lower and upper boundaries of the QTL. Cluster shows the mean cluster the most significant phenotype belongs to .Candidate gene shows our best guess as to what gene is causing this QTL. Arabidopsis Locus shows the Arabidopsis equivalent which can be found in the TAIR database. Source shows what paper first described this gene or locus, if available.

**Supplementary Table 9: QTLs.all. Prominent loci found when using the extended descriptives.** All prominent loci as defined in the paper and shown in supplemental figure with QTLs for all extended descriptives. The phenotype column shows the phenotype with the most significant result. Phenotypes show all the phenotypes that have a significant result at the location of the most significant SNP. Position shows the position of the most significant SNP in Mbp. Pval shows the -log(pvalue). l.bound and u.bound show the lower and upper boundaries of the QTL. Cluster shows the all_cluster the most significant phenotype belongs to. Candidate gene shows our best guess as to what gene is causing this QTL. Arabidopsis Locus shows the Arabidopsis equivalent which can be found in the TAIR database. Source shows what paper first described this gene or locus, if available.

**Supplementary Table 10: Heritability. Heritability scores of all traits.** Broad-sense heritability for each trait was calculated by taking ratio of the between genotype variance and the total variance.

**Supplementary Table 11: Correlation matrix. Correlation of all traits with each other from 0 to 1.** Correlation matrix of all traits. Created with the cor() function in base R. For interpreation of the names see the Pheno_definitions and Statistical_definitions sheet.

**Supplementary Table 12: LD_decay. Linkage Disequillibrium Decay.** Summary of linkage disequillibrium for each chromosome.

**Supplementary Table 13: pvalues. pvalues of all significant (-log10 >7) SNPs after GWAS of all phenotypes.** All significant SNPs for all traits. Phenotype shows the phenotype, for interpretation of the names see pheno_definitions and statistical_defintions. Position is the position of the SNP in Mbp. Pval is the -log10(pvalue). Mean_clustering shows what cluster this trait belongs to in the mean clustering, figure 3 and 5 in the paper. All_clustering shows what cluster this trait belongs when we do clustering on all traits, as shown in supplemental figure 8.

**Supplementary Table 14: Height.mean.day2. GWAS result on height for generating figure 4.** GWAS results for height on day-93. pval is the -log10(p-value) for each SNP, chr is the chromosome, Position is the Position in Mbp,zscore shows the relation to the mean of that phenotype, snp_index shows what number that SNP is

**Supplementary Table 15: gbrat.mean.day2. GWAS result on green blue ratio for generating figure 4.** GWAS results for the green blue ratio on day-93. pval is the -log10(p-value) for each SNP, chr is the chromosome, Position is the Position in Mbp ), zscore shows the relation to the mean of that phenotype, snp_index shows what number that SNP is

**Supplementary Table 16: Rededge.mean.day2. GWAS result on red.edge for generating figure 4.** GWAS results for the rededge on day-93. pval is the -log10(p-value) for each SNP, chr is the chromosome, Position is the Position in Mbp, zscore shows the relation to the mean of that phenotype, snp_index shows what number that SNP is

**Supplementary Table 17: Height.traits. All height traits in one sheet.** The GWAS results for all height traits in 1 sheet. As shown in figure 6.
